# iModEst: disentangling -omic impacts on gene expression variation across genes and tissues

**DOI:** 10.1093/nargab/lqaf011

**Published:** 2025-03-04

**Authors:** Dustin J Sokolowski, Mingjie Mai, Arnav Verma, Gabriela Morgenshtern, Vallijah Subasri, Hareem Naveed, Maria Yampolsky, Michael D Wilson, Anna Goldenberg, Lauren Erdman

**Affiliations:** Department of Molecular Genetics, University of Toronto, ON M5S 3K3, Canada; Department of Computer Science, University of Toronto, ON M5S 2E4, Canada; Department of Computer Science, University of Toronto, ON M5S 2E4, Canada; SickKids Research Institute, Program in Genetics and Genome Biology, ON M5G 0A4, Canada; Vector Institute; Department of Computer Science, University of Toronto, ON M5S 2E4, Canada; Department of Computer Science, University of Toronto, ON M5S 2E4, Canada; SickKids Research Institute, Program in Genetics and Genome Biology, ON M5G 0A4, Canada; Vector Institute; SickKids Research Institute, Program in Genetics and Genome Biology, ON M5G 0A4, Canada; Department of Medical Biophysics, University of Toronto, ON M5G 2C4, Canada; Department of Computer Science, University of Toronto, ON M5S 2E4, Canada; SickKids Research Institute, Program in Genetics and Genome Biology, ON M5G 0A4, Canada; SickKids Research Institute, Program in Genetics and Genome Biology, ON M5G 0A4, Canada; Department of Molecular Genetics, University of Toronto, ON M5S 3K3, Canada; SickKids Research Institute, Program in Genetics and Genome Biology, ON M5G 0A4, Canada; Department of Computer Science, University of Toronto, ON M5S 2E4, Canada; SickKids Research Institute, Program in Genetics and Genome Biology, ON M5G 0A4, Canada; Vector Institute; CIFAR: Child and Brain Development, Toronto, ON M5G 1M1, Canada; Department of Computer Science, University of Toronto, ON M5S 2E4, Canada; SickKids Research Institute, Program in Genetics and Genome Biology, ON M5G 0A4, Canada; Vector Institute; James M. Anderson Center for Health Systems Excellence, Cincinnati Children’s Hospital Medical Center, Cincinnati, OH 45229, USA; College of Medicine, University of Cincinnati, OH 45267, United States

## Abstract

Many regulatory factors impact the expression of individual genes including, but not limited, to microRNA, long non-coding RNA (lncRNA), transcription factors (TFs), *cis-*methylation, copy number variation (CNV), and single-nucleotide polymorphisms (SNPs). While each mechanism can influence gene expression substantially, the relative importance of each mechanism at the level of individual genes and tissues is poorly understood. Here, we present the integrative Models of Estimated gene expression (iModEst), which details the relative contribution of different regulators to the gene expression of 16,000 genes and 21 tissues within The Cancer Genome Atlas (TCGA). Specifically, we derive predictive models of gene expression using tumour data and test their predictive accuracy in cancerous and tumour-adjacent tissues. Our models can explain up to 70% of the variance in gene expression across 43% of the genes within both tumour and tumour-adjacent tissues. We confirm that TF expression best predicts gene expression in both tumour and tumour-adjacent tissue whereas methylation predictive models in tumour tissues does not transfer well to tumour adjacent tissues. We find new patterns and recapitulate previously reported relationships between regulator and gene-expression, such as CNV-predicted *FGFR2* expression and SNP-predicted *TP63* expression. Together, iModEst offers an interactive, comprehensive atlas of individual regulator–gene–tissue expression relationships as well as relationships between regulators.

## Graphical Abstract

**Graphical Abstract:**
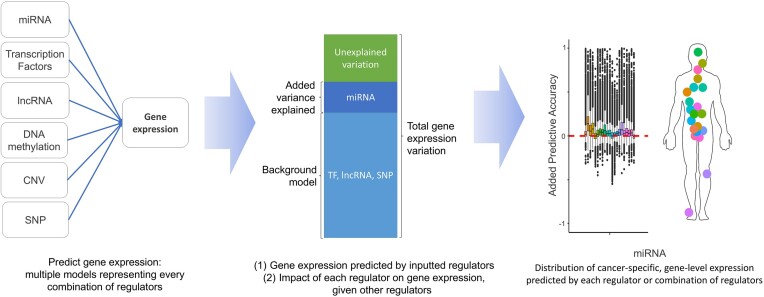

## Introduction

The precise regulation of gene expression is crucial for proper cellular function in all organisms. Gene expression can be clustered into cellular networks whose expression is controlled by a series of regulatory molecules, many of which are encoded by genes themselves [1]. The level at which a gene is expressed can drive cell fates and cell states (e.g. inflammatory activation) and gene expression levels can be a by-product of these cellular events [1]. Accordingly, gene expression is also dynamic to the environment of the cell and the individual. These interacting factors make investigating how genes are regulated in a high-throughput manner challenging; however, applying large-scale multi-omics approaches allows researchers to generate robust associations between DNA sequences, regulatory molecules (e.g. DNA methylation status), and gene expression, thereby allowing us to take a multi-omic approach in systematically profiling patterns of gene expression and regulation [2–7].

Large-scale multi-omics approaches that systematically interrogate gene expression and regulation require a massive volume of high-quality data. Large genome-wide sequencing data repositories, such as The Cancer Genome Atlas (TCGA), Encyclopedia of DNA elements (ENCODE), and the Gene Tissue Expression (GTEx) project, provide an opportunity to integrate multi-omic data across hundreds of individuals, facilitating highly powered gene-regulatory analysis [5, 8–18]. For example, many cross-tissue, multi-omic analyses have been performed using TCGA, resulting in the identification of distinct immunological subtypes across tissues, unique genomic network patterns in squamous carcinomas, and a global increase in methylation and aneuploidy in cancer relative to normal or tumour-adjacent tissues [5, 9, 19, 20, 21]. These datasets also provide an unprecedented opportunity to profile the gene regulatory mechanisms within genes that are not commonly studied [22]. GTEx and TCGA perform these multi-omic screens for every tissue sample collected (e.g. RNA-seq, DNA methlyation, and whole genome sequencing of the same sample), allowing researchers to perform paired analyses across regulatory modalities.

The GTEx consortium computed expression quantitative trait loci (eQTLs), characterizing the relationship between DNA sequence variation and gene expression variation across tissues, and found that the majority of genes are affected by local genetic variation but a number of important distal relationships between genetic variation and gene expression also exist [14]. These results demonstrate that *cis-*eQTLs are a reliable starting-point for predicting gene expression at a genome-wide level. In addition to genetic variation, other regulatory features [e.g. miRNA, long non-coding RNA (lncRNA), transcription factors (TFs), and DNA methylation] control gene expression at the transcriptional and post-transcriptional level. Studies investigating gene regulation within TCGA data often rely on somatic variants in the tumour, which have contributed to our understanding of disease [23–25]. In contrast, studying the germline genetic variations available in TCGA may be more generalizable to investigating gene expression in a non-cancer setting [23, 24, 26–28]. Together, these previous studies in combination with the multi-omic data within TCGA provides the opportunity to build and evaluate an unbiased profile of how multiple gene-regulatory modalities regulate the expression of every gene in dozens of human tumour and normal (tumour-adjacent) tissues.

**Figure 1.**
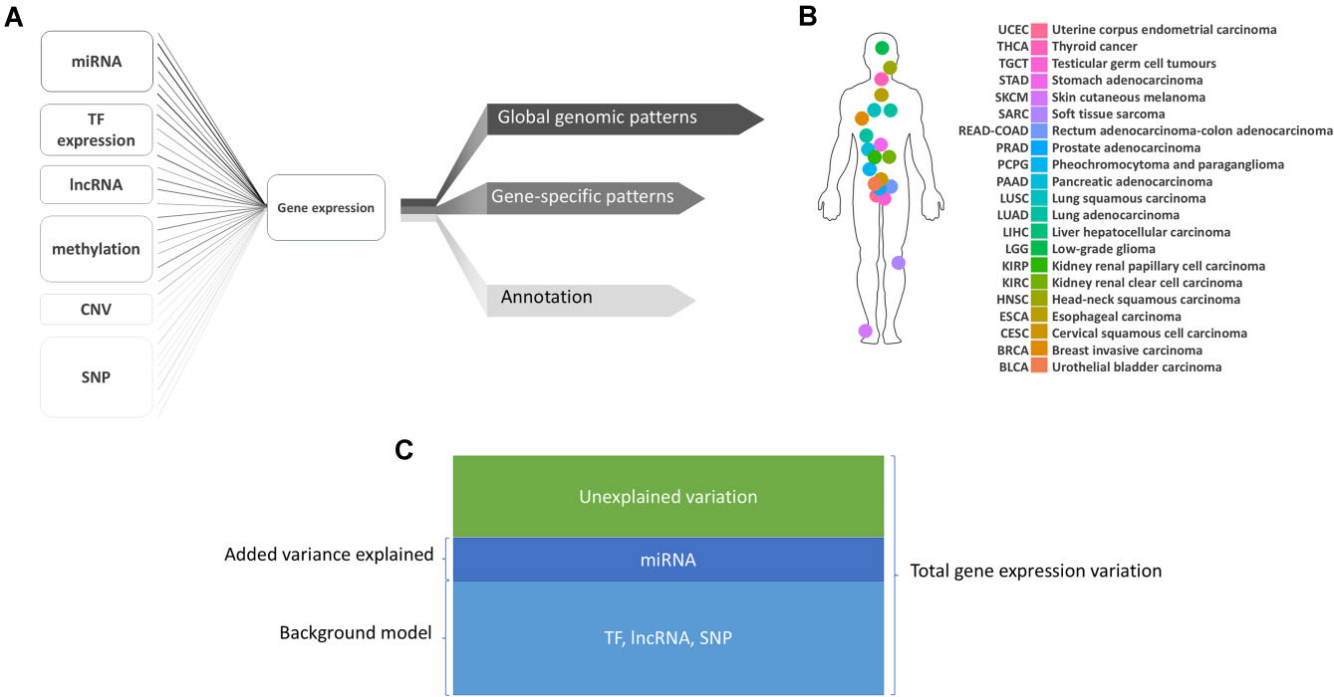
Summary of iModEst data input and output (**A**) iModEst gene expression model. (**B**) TCGA tissues assessed independently in iModEst. (**C**) Schema of gene expression variation explained by miRNA in a given model.

In this work, we elucidate patterns of gene expression regulation by building models to predict each gene’s expression separately in 21 TCGA cancer tissues (sample sizes: Supplementary Table S1) using combinations of the regulators collected in TCGA: miRNA, lncRNA, methylation, single-nucleotide polymorphisms (SNPs), TFs, and copy number variations (CNVs) (Fig. 1A and B). Overall, we learn 63 models per gene, a total of 13,929,216 models across all genes and tissues, allowing us to measure the relative gain in predictive accuracy per regulator type, gene, and tissue. We find that for 43% of the >16,000 genes that we considered, our tumour-derived models are able to explain up to 70% of gene expression variance in both tumour and tumour-adjacent tissues. These models further highlight the value of taking an integrative approach to studying gene expression regulation because we predict gene expression at a genome-wide scale more consistently than any other approach thus far [18, 29].

## Materials and methods

### Ethical compliance

Access to the data for this study was acquired through the Database of Genomes and Phenotypes (DBGaP) with institutional Research Ethics Board approval from the Hospital for Sick Children.

### Sample acquisition and preprocessing

Quantified miRNA (level 3), RNA-seq fastq files (level 1), SNP CEL files (level 1), methylation beta-value files (level 3), and clinical data files (level 3) were downloaded for 26 cancers from TCGA. These data were preprocessed and screened for sample size (>100 unrelated samples across all data types), resulting in a final set of 21 cancers, with 4 removed for insufficient sample in at least one data type and READ and COAD combined and analyzed as a single cancer (Supplementary Fig. S1). Preprocessing was performed for each cancer separately and is described below. For a sample to be included in iModEst, they needed to have dbGap-accessible RNA-seq data, miRNA-seq data, somatic CNV data, and germline SNP data. Samples with all of these data allow us to pair our models across regulators. Each dataset must pass quality control for every datatype. Lastly, they must have clinical data available.

### RNA-seq

RNA-Seq HiSeq paired-end sequencing raw reads (FASTQ) were downloaded from GDC (https://portal.gdc.cancer.gov/). A Salmon quasi mapping index was built based on gencode.v19.protein-coding_transcript and gencode.v19.lncRNA_transcript [30]. Trim_galore/0.4.4 was then used to do quality trimming (Phred quality score threshold of 17) and adapter trimming. Short paired-end reads were then removed (threshold 17 bps) [https://github.com/FelixKrueger/TrimGalore/blob/master/Docs/Trim_Galore_User_Guide.md]. Finally, gene expression was quantified from processed paired-end RNA-seq data, together with sequence-specific bias correction and fragment GC bias correction, using salmon/0.82 [31].

The resulting gene expression values were first standardized (log transformed, subtracted mean, divided by standard deviation; performed per gene, per cancer), and then corrected for gender, batch, the first two principal components from germline SNP data [32], percentage of tumour nuclei, age at initial pathological diagnosis, ICD-10, ICD-O-3 histology, and histological type in a linear model. Finally, these values were standardized per gene again. Normal TCGA samples were corrected for gender, batch, and the first two principal components from the SNP data in normal TCGA samples in a separate linear model.

### miRNA

miRNA isoform data were downloaded from GDC from Firebrowse (TCGA data version 2016_01_28). Normalized reads for each miRNA sequence isoform observed per sample were acquired using the BCGSC miRNA profiling pipeline [33]. miRNA expression in cancer samples was first standardized per miRNA, and then corrected for gender, batch, the first two principal components from germline SNP data, percentage of tumour nuclei, age at initial pathological diagnosis, ICD-10, ICD-O-3 histology, and histological type in a linear model. These values were standardized again per miRNA. miRNA expression for normal TCGA samples was corrected for gender, batch, and the first two principal components from the SNP data in normal TCGA samples in a separate linear model.

### Methylation

Normalized methylation values by probe from the Illumina Infinium HumanMethylation450 BeadChip array were obtained from Firebrowse (TCGA data version 2016_01_28). Probe beta-values were converted to *M*-values. Methylation *M*-values in cancer samples were first standardized per probe and then corrected for gender, batch, the first two principal components from germline SNP data, percentage of tumour nuclei, age at initial pathological diagnosis, ICD-10, ICD-O-3 histology, and histological type in a linear model. These values were standardized again by probe. Methylation *M*-values for normal TCGA samples were corrected for gender, batch, and the first two principal components from the germline SNP data in a separate linear model. Probes located within ±1 MB of a gene’s boundary were considered annotated to the given gene and used as predictors for that gene.

### Copy number variation

Gene copy numbers were obtained from Firebrowse (TCGA data version 2016_01_28), generated from the Affymetrix SNP6 array [34]. CNV values were first normalized per gene by subtracting the mean and dividing by the standard deviation. Then, normalized CNV values in cancer samples were corrected for gender, batch, the first two principal components from germline SNP data, percentage of tumour nuclei, age at initial pathological diagnosis, ICD-10, ICD-O-3 histology, and histological type in a linear model. These values are normalized again for each gene. Normalized CNV values for normal TCGA samples were corrected for gender, batch, and the first two principal components from the germline SNP data in a separate linear model.

### Single-nucleotide polymorphisms

SNP array quality control (pipeline: Supplementary Fig. S2): Affymetrix array .CEL files were acquired from GDC and converted to PLINK PED and MAP files using birdseed version 2 from Birdsuite version 1.5.5 [35]. Quality control was performed on the resulting files using PLINK version 1.9. Quality control removed SNPs with a call rate <0.1 and individuals with a call rate <0.05, SNPs with a Hardy–Weinberg chi-square *P*-value <1e-05, individuals with ambiguous sex, and individuals who are related as first cousins or closer (PLINK PI_HAT ≥ 0.12) [36].

Controlling for ethnic admixture: SNP data were merged with a subset of the 1000 Genomes data set (CEU, GBR, TSI, IBS, and FIN population individuals, a white, European set) from the Illumina Omni2.5M chip [37] to identify individuals in our sample who are ethnic outliers relative to these populations. Overlapping SNPs between the data sets were retained and SNPs that PLINK detected as strand errors between the two were removed. To extract independent SNPs which can be used to compute sample relations, the merged file was pruned using a sliding window of size 1500 bases, moving 100 bases at a time, detecting SNPs with a pairwise *r*^2^ of 0.2 or greater, and removing them. Principal component analysis was then conducted on the pruned SNP data using a combination of the KING software suite and the PC-Air method contained in the GENESIS R package (version 2.34.0) [32, 38]. Population outliers were identified as observations that were more than a factor of 6 standard deviations from the mean of any of the five specified 1000 Genomes population groups (CEU, GBR, TSI, IBS, and FIN) along the first five principal components. Population outliers were first identified and removed (Supplementary File S1). Then, principal components for the sample alone and sample outliers, defined as observations that were more than a factor of 6 standard deviations away from the mean along any one of the first five principal components, were identified and removed. Finally, principal components were computed one last time in the final sample and the top 2 principal components, as suggested by the PC-Air paper, were used to control for any additional ethnic admixture in the data [32].

Imputing SNP array data: The autosomes of the sample that remained after the principal component analysis were then pre-phased using SHAPEIT version 2.790 and 1000 Genomes phase 3 as reference files [39]. The pre-phased autosomes were then imputed using IMPUTE22 version 2.3.1, again using 1000 Genomes phase 3 as reference data [40]. The imputed data underwent further quality control by removing SNPs with a missingness rate >0.05, minor allele frequency <0.05, imputation information level <0.8, and a Hardy–Weinberg chi-square *P*-value <1e-20. SNPs that passed this quality control were then adjusted to incorporate somatic changes in the imputed SNPs that were retained based on variant calls from Ellrott *et al.* [41] and converted to dosage calls. SNPs were annotated to genes based on their proximity ±1 MB from the gene boundary, as was done in the GTEx Consortium [42].

### Statistical analyses

#### Predictive model training and testing

Across these 21 cancers, we built per-gene and per-cancer predictive models by fitting an elastic net model [43] with mixing parameter 0.5 using all possible combinations of miRNA, TFs, lncRNA, methylation CpG sites, CNV, and eQTLs as predictors. Our elastic net model was fit using the glmnet v2.0-12 [44] implementation of elastic net in R version 3.3.2. We included miRNA and TFs in our models based on evidence of them targeting the gene of interest from TargetScan (6-, 7-, and 8-mer matching) and ENCODE, respectively [16, 45]. Specifically, we used ENCODE TF–gene interactions and miRNA–gene interaction dataset as a universe to connect genes to regulators. For example, TF prediction of gene *A* was only computed from the TFs that have been shown to bind to gene *A* in ENCODE. Methylation probes, lncRNA, and SNPs within 1 MB of the gene were included in gene models. Furthermore, lncRNA within the transcript body was excluded to avoid including genes being predicted by their own lncRNA. We measured out-of-sample predictive accuracy based on leave-one-out predictive residual error sum of squares (PRESS R^2^), (PRESS R^2^= 1 − SSE/TSS). Intuitively, this represents the error variance in held out samples normalized by the variance of the data.

#### PRESS R2 deflation

In order to account for inflation in the PRESS R^2^ values due to leave-one-out cross validation in a sample that has been normalized with our training data, we calculated PRESS R^2^ deflation values by generating random predictions for each individual drawn from a normal distribution based on the mean and standard deviation of the sample without that individual. We then calculated the PRESS R^2^ statistics from these random predictions over 10 000 runs and used the average of these as our PRESS R^2^ deflation value. This was done for each gene in each cancer. Deflation values >0 were then subtracted from the PRESS R^2^ value derived from each model of the corresponding gene and cancer.

#### Regulator evaluation

The most predictive regulators of a gene's expression were evaluated using the gain in predictive accuracy (average increase in PRESS R^2^ across all background models) when that regulator type (i.e. miRNA, TF expression, lncRNA, methylation, and CNV) was added to the predictive model. Therefore, the predictive gain from miRNA for a given gene is calculated based on the average change in accuracy from all the models without miRNA to all models with miRNA.

#### Coefficient evaluation

Within our groups of expression regulators, we evaluated their relative importance on a per-gene and per-cancer basis by calculating their average effect in the 32 models they were included in. Because our number of samples is fewer than our number of features (n << p), we modeled our data using an elastic net regularized regression [43] with a 0.5 mixing parameter. In this context, the coefficient direction is stable and therefore interpretable but the size can be impacted both by shrinkage (due to regularization) and correlation structure in the predictors. Therefore our approach tends to upweight more independent regulators yet will still show a contributory effect from correlated regulators.

#### Gene clustering

Clustering was conducted over the genes in each cancer independently. The 63 models computed for each gene and cancer were the features used in clustering. Genes in the 21 cancers were subset to only those expressed in every cancer. A first stage of clustering was conducted across genes for each cancer independently based on PRESS R2 scores. We selected the number of gene clusters by choosing the maximum number of clusters produced by any cancer based on connectivity and Dunn index in the clValid package version 0.6-6 [46]. Then, we generated 20 clusters for each cancer then labeled them based on the top regulator(s) in the cluster. Top regulators were determined by identifying regulators that added the most explanatory variance to the genes in a given cluster. All regulators that contributed at least half the gain in predictive accuracy as the top regulator were included in the cluster label. Final clusters of genes were then determined by grouping clusters of genes based on their regulator label(s).

## Results

### Exploring latent biological underpinnings driving predictions in iModEst

iModEst associates variation in combinations of regulatory molecules with variation in gene expression to predict which groups of regulatory molecules regulate each gene in each cancer. Briefly, iModEst functions as an interactive database through a web application, where users input a gene of interest and a model (i.e. whether they want each regulator to be included, excluded, or controlled for) (Supplementary File S2). iModEst outputs which class of regulatory modalities (e.g. TFs, miRNA, etc.) most strongly covaries with their genes’ expression given the user-selected models across each of the 21 cancers in iModEst. These outputs are presented as a change in PRESS R^2^ values, which represent the degree of predictive gain by each mode on the expression of the selected gene. As such, an output of >0 for miRNA can be interpreted miRNAs positively contributing to the explanation of the variability of gene expression in that gene and cancer. In contrast, an output of 0 (or less), reflects that including miRNA in the model reduces the model’s ability to predict gene expression for that gene and cancer. iModEst also outputs which specific regulatory groups (e.g. TFs, miRNA, and *cis-*SNPs) are driving gene expression prediction as well as further zooming in to particularly influential regulators within these groups.

**Figure 2.**
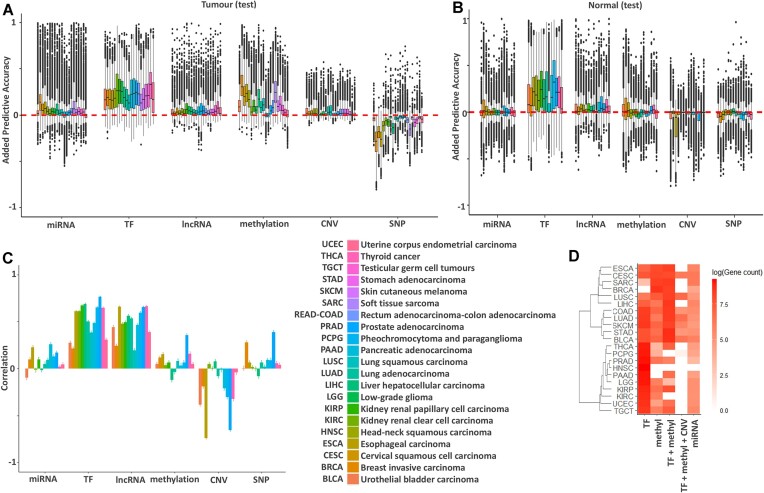
Summary of per-regulator impact on gene expression prediction. (**A**) Average change in accuracy (PRESS R^2^) of models predicting gene expression as each of 6 different regulator groups are added to models with all possible combinations of the remaining regulators (32 combinations). This graph shows data for 10,364 genes expressed in all tumour tissues. (**B**) Average change in accuracy (PRESS R^2^) of models from panel (A) applied to tumour-adjacent tissue adjacent to each cancer. In tumour-adjacent samples, TFs remain predictive whereas the added explanatory variance from methylation drops dramatically. Note that tumour-adjacent samples were only available in a subset of cancers. (**C**) Pearson correlation between per-gene model accuracy gains in tumour versus tumour-adjacent tissue. (**D**) Logged count of genes best predicted by each regulator set based on clustering results. The top 5 regulator clustering sets and the logged number of genes best predicted by them, averaged over 21 cancers, are shown with the logged number of genes indicated by color bar transparency. Cancers are ordered by regulatory similarity on the *y*-axis.

We investigated the influence of tumour-specific regulation on gene expression predictions by investigating the difference between the predictive gain of each regulatory modality between tumour tissues and tumour-adjacent tissues in each gene and tumour type. Models were trained on tumour tissues and applied to tumour-adjacent tissues, meaning that if a model could not be transferred to the tumour-adjacent tissue, the more cancer-specific our regulatory predictions were (Fig. 2C and Supplementary File S3). Overall, we found three groups of gene–regulator pairs. The largest group of gene–regulator pairs were those with non-zero predictions in both tumour and tumour-adjacent tissues (median percent of genes that transfer between tissues: miRNA = 40.49%, TF = 79.78%, lncRNA = 66.38%, DNAm = 38.48%, CNV = 33.56%, SNP = 3.20%).

Overall, we found that gene–regulator pairs that transferred to tumour-adjacent tissues had a similar predictive gain in both tissues (median difference in predictive gain: miRNA = 0.0804%, TF = 3.13%, lncRNA = 1.15%, DNAm = 3.47%, CNV = 0.0193%, SNP = 2.17%), suggesting these predictions reflect putative regulatory patterns. The second most common set of gene–regulator pairs were those that did not transfer to tumour-adjacent tissues and are therefore cancer-specific regulatory predictions (median percent of tumour-specific regulatory predictions: miRNA = 45.60%, TF = 20.78%, lncRNA = 29.07%, DNAm = 50.90%, CNV = 55.77%, SNP = 9.38%). The least common set of gene-regulator pairs were those that were not predictive in tumour tissues but were predictive in tumour-adjacent tissues (median percent tumour-adjacent specific regulatory predictions: miRNA = 12.07%, TF = 2.38%, lncRNA = 7.94%, DNAm = 7.45%, CNV = 11.68%, SNP = 85.57%). These tumour-adjacent predicted gene-regulator pairs likely represent genes where a strong cancer-specific signal diminishes a tumour-adjacent signal. For example, the high proportion of tumour-adjacent gene-SNP pairs likely represents a weak eQTL signal that is diminished by a strong cancer signal in the tumour data.

Next, we explored where tissue-specific regulation contributed to regulator prediction in iModEst. For example, TFs play an extremely important role in determining cell fate. As such, if the regulatory predictions in iModEst are driven by cellular composition, then we would expect genes with cell-type-specific gene expression to be regulated by TFs. We tested this by pooling cell-type markers identified from single-cell RNA-seq datasets in paraganglioma and pheochromocytoma (PCPG) [47] and breast cancer (BRCA) [48] and asking whether genes that are predicted by each regulatory molecule by >25% are over-represented in these cell-type markers using an unranked hypergeometric test [49]. We found that miRNAs were the only regulator that was over-represented in cell-type markers in either PCPG (FDR-adjusted *P*-value 4.32 × 10^-7^, 21 genes) or BRCA (FDR-adjusted *P*-value = 1.87 × 10^-10^, 166 genes). We also identified specific cell types to be over-represented for miRNA-, methylation-, TF-, and lncRNA- regulated genes; however, these cell-type specific signatures were weaker (Supplementary Fig. S3). Accordingly, the predictions in iModEst are not linked to TF-driven cell-type specific regulation, but some of the underlying predictions may be related to miRNA-driven cell-type specific gene expression.

Last, individual genomic regions may be influenced by multiple regulatory factors. For example, a SNP found in the promoter region of a gene may impact a CpG site and a TF-binding site. In this example, iModEst would include the SNP, the CpG site, and the expression of the associated TF within their models; however, our stringent regularization and prior-independent model building (i.e. a SNP alters a CpG which impacts TF binding) algorithm prevents us from inferring specific regulator–regulator interactions. We did not include these model interactions because the directionality of these interactions is often ambiguous (e.g. TF influencing miRNA or miRNA influencing TF). Further, each regulator’s predictions are an accumulation of multiple regulatory molecules (i.e. all gene-binding TFs, all gene-binding miRNA, SNPs, lncRNAs, and CpGs within a 1 Mb window of the gene). Accordingly, these models do not reflect a single regulatory interaction but an accumulation of regulatory interactions across the gene. For example, we identified genes where SNPs mediate miRNAs in our models, which could reflect mirSNPs. These genes did not significantly enrich previously discovered mirSNPs in the TCGA database [50]. As such, predictions in iModEst should be considered a gene-level categorization of the major regulatory classes that influence the expression of that gene.

### Overview of iModEst’s development and functionality

The influence of germline DNA sequence variability on gene expression (eQTL) is mediated by the regulatory modalities (e.g. TF interactions with a binding motif, miRNA binding, and DNA methylation) these DNA sequences impact. Similarly, genes are grouped within co-expression networks, meaning that gene expression is variable independent of DNA sequence variability within the gene (e.g. changes in TF interactions and DNA methylation due to external stimulus). TCGA contains publicly available RNA sequencing, miRNA isoform capture [33], DNA methylation arrays, gene copy numbers, and SNP arrays across over 10,000 samples spanning 21 tumour times. These data allowed us to develop a framework that lets us systematically profile the association between one or multiple regulatory molecules in a high-throughput manner. With our framework, we modeled the association between six regulatory modalities that could be extracted from these data (TFs, methylation, lncRNA, miRNA, SNPs, and CNVs) on the expression of 16,000 genes across 21 cancers (63 models per gene, 13 929 216 models total).

We developed iModEst with the goal of avoiding overfitting and over-predicting our models as much as possible. These over-predictions were most likely to arise from three sources: (i) the high ratio of features to samples, (ii) the noise inherent to multi-omic data types (particularly SNP variation and DNA methylation), and (iii) the phenomena of better studied genes having more publicly available data and annotations. We accounted for the high ratio of features compared to samples by correcting for population-level data using a linear regression (gender, age, batch, and ancestry-related SNPs), and tumour-class data (ICD-10, ICD-O-3 histology, and histological type) rather than stratifying our data by these factors, regularizing our models using elastic net, and testing our models in held out samples. These corrections allowed us to maximize our total sample size and reduce overfitting at the cost of biological variability that has been shown to impact gene expression in this study (Supplementary Fig. S4) and others [51–53]. Similarly, we limited our data sources to those that are present in 100 samples across each cancer, with the exception of two borderline cases of UCEC (*n* = 90) and BRCA (*n* = 99) (e.g. excluding ATAC-seq, snRNA-seq that are present in certain samples in certain cancers). Due to our use of the elastic net model, coefficient direction is stable and therefore interpretable but the size can be impacted both by shrinkage (due to regularization) and correlation structure in the predictors. Therefore our approach tends to upweight more independent regulators yet will still show contributory effects from correlated regulators (i.e. downweighting regulator–regulator interactions). Last, we accounted for the phenomena where more “famous” genes have more resources by limiting our modality-gene evidence to gene-proximity and high-throughput assays that are available for every gene in every cancer. Together, the predictions in iModEst should be considered a conservative estimate of how the variability of TFs, methylation, lncRNA, miRNA, SNPs, and CNVs influence gene expression in the TCGA database.

Explanatory variance from TFs for a gene of interest was completed by associating co-expression from TFs that have been shown to bind to the gene’s promoter, based on ENCODE’s list of TF–gene associations [16, 54]. For methylation, explanatory variance is obtained by statistically associating gene expression to DNA methylation levels 1 megabase (Mb) around the gene TSS. For lncRNA, explanatory variance of a gene was measured by associating the gene expression of a lncRNA within 1 Mb of the gene to the expression of the gene. lncRNAs that directly overlap with the gene (e.g. lncRNA on the opposite strand or in an intron of the gene of interest) were excluded to prevent artificial inflation of explanatory variance. For miRNA, explanatory variance for a gene of interest is obtained by associating miRNA expression binding to the gene’s 3′UTR using ENCODE’s list of miRNA-gene binding associations [16, 45]. For CNVs, explanatory variance for a gene is obtained by associating whole-gene CNVs. Finally, for SNPs, explanatory variance for a gene is obtained by associating common (>5% of the population) germline variants to gene expression.

We quantified our models’ predictive accuracy by iteratively leaving one sample out of our predictive models and calculating the predicted residual error sum of squares (PRESS) R^2^ statistic [55]. We then calculated the gain in predictive accuracy per regulator by averaging the PRESS R^2^ increase compared to the average PRESS R^2^ of the background models without the regulator of interest. For example, the average PRESS R^2^ gain when miRNA is added to a model with no regulators, a model with TFs, a model with TFs and lncRNA, a model with TFs, lncRNA, methylation, etc. We measure the importance that a regulator may have on gene expression by quantifying the decrease in predictive accuracy after that regulator is removed. This metric can therefore be thought of as a type of group-wise Shapley value since the additive impact is across a set of predictors, rather than a single predictor [56]. As such, if we state that TFs predict 25% of a gene’s expression, then removing TFs from the model will on average decrease the predictive accuracy by 25%. To ensure that these estimates aren’t inflated by a random signal in the data, deflation statistics were computed for each cancer by generating 1000 PRESS R^2^ values based on random gene expression predictions, simulated based on samples from a normal distribution for each gene in each cancer. These simulations showed that random PRESS R^2^ values were always <0 (Supplementary Fig. S5) thus PRESS R^2^ values > 0 can be interpreted as a robust metric of predictive variance explained.

We find the presence of TFs in our models showed the largest gain in predictive accuracy for the majority of the genes across cancers except for genes in breast (BRCA), cervical (CESC) and esophageal cancers (ESCA) as well as soft-tissue sarcoma (SARC) [14, 57, 58] where methylation data were found to be the most predictive (Table 1). We benchmarked our results by showing that they reveal strong regulatory effects already known in well-studied genes, such as CNV regulation of *KRAS* and *MDM2*. Since this atlas is the first of its kind, we could only benchmark the predictive accuracy of our models on a case-by-case basis. We turned to COSMIC genes because they are well annotated and found that our regulatory predictions accurately predict published work. We then applied our results to relatively lesser-known genes and described novel strong regulatory effects such as eQTL regulation in *CRYBB2*.

**Table 1.** Average contribution [mean (median)] to variance explained by each regulator in each cancer in both tumour and tumour-adjacent tissue

	microRNA		Transcription factors		Long non-coding RNA		Methylation		Copy number variation		Expression quantitative trait loci	
Cancer	Tumour	Normal	Tumour	Normal	Tumour	Normal	Tumour	Normal	Tumour	Normal	Tumour	Normal
BLCA	4% (2%)	−0.01% (0%)	19% (17%)	0.15% (0.08%)	4% (2%)	0.05% (0.01%)	11% (9%)	0.02% (0%)	5% (2%)	−0.04% (0%)	−4% (−4%)	−0.03% (−0.01%)
BRCA	15% (14%)	0.04% (0.02%)	19% (19%)	0.12% (0.07%)	3% (2%)	0.02% (0.01%)	34% (31%)	0.04% (0.01%)	3% (1%)	−0.01% (0%)	−29% (−28%)	−0.06% (−0.04%)
CESC	8% (5%)	–	19% (18%)	–	5% (3%)	–	22% (20%)	–	3% (1%)	–	−19% (−18%)	–
ESCA	10% (8%)	0.02% (0.01%)	18% (17%)	0.24% (0.22%)	4% (2%)	0.06% (0.03%)	25% (23%)	0.03% (0.01%)	4% (2%)	−0.15% (−0.06%)	−27% (−26%)	−0.04% (−0.03%)
HNSC	0% (0%)	0% (0%)	22% (18%)	0.23% (0.18%)	4% (2%)	0.05% (0.02%)	−1% (−1%)	−0.01% (−0.01%)	−2% (0%)	−0.02% (0%)	0% (0%)	0% (0%)
KIRC	4% (2%)	−0.01% (0%)	29% (28%)	0.19% (0.12%)	4% (2%)	0.02% (0%)	6% (3%)	0% (0%)	1% (0%)	0% (0%)	−7% (−6%)	0% (0%)
KIRP	6% (4%)	0.01% (0%)	27% (28%)	0.26% (0.24%)	8% (7%)	0.05% (0.02%)	12% (9%)	−0.01% (−0.01%)	2% (1%)	0% (0%)	−9% (−8%)	0% (0%)
LGG	5% (4%)	–	26% (26%)	–	7% (5%)	–	7% (5%)	–	3% (1%)	–	−2% (-2%)	–
LIHC	8% (6%)	0% (0%)	21% (20%)	0.2% (0.14%)	6% (4%)	0.04% (0.02%)	19% (17%)	−0.03% (-0.02%)	1% (0%)	0% (0%)	−15% (−14%)	0.01% (0.01%)
LUAD	4% (2%)	0% (0%)	19% (17%)	0.23% (0.16%)	4% (3%)	0.05% (0.01%)	10% (8%)	−0.01% (0%)	6% (3%)	0% (0%)	−3% (−3%)	-0.01% (−0.01%)
LUSC	4% (2%)	0% (0%)	15% (13%)	0.07% (0%)	4% (2%)	0.01% (0%)	13% (11%)	0.01% (0%)	6% (4%)	0% (0%)	−5% (−4%)	−0.01% (0%)
PAAD	0% (0%)	0.02% (0%)	25% (23%)	0.21% (0.16%)	6% (4%)	0.06% (0.02%)	1% (0%)	−0.02% (-0.01%)	2% (0%)	−0.02% (0%)	−1% (−1%)	−0.03% (−0.02%)
PCPG	2% (1%)	0.01% (0%)	26% (24%)	0.32% (0.32%)	8% (5%)	0.09% (0.04%)	1% (0%)	−0.01% (−0.01%)	2% (0%)	−0.01% (0%)	−2% (−2%)	−0.04% (−0.03%)
PRAD	3% (2%)	0.01% (0%)	26% (25%)	0.23% (0.21%)	7% (5%)	0.06% (0.03%)	7% (5%)	0.03% (0.01%)	3% (1%)	−0.06% (−0.01%)	−2% (−2%)	−0.01% (−0.01%)
READ-												
COAD	5% (3%)	–	24% (23%)	–	5% (3%)	–	11% (8%)	–	4% (2%)	–	−6% (−5%)	–
SARC	11% (7%)	–	15% (14%)	–	3% (2%)	–	27% (25%)	–	4% (2%)	–	−16% (−15%)	–
SKCM	4% (2%)	–	19% (18%)	–	5% (3%)	–	11% (9%)	–	4% (2%)	–	−3% (−3%)	–
STAD	6% (4%)	–	22% (22%)	–	5% (3%)	–	14% (12%)	–	4% (2%)	–	−9% (−8%)	–
TGCT	4% (2%)	–	23% (22%)	–	7% (5%)	–	7% (5%)	–	3% (1%)	–	−6% (−5%)	–
THCA	5% (3%)	0.01% (0%)	33% (34%)	0.23% (0.21%)	10% (8%)	0.08% (0.05%)	4% (2%)	0.03% (0.02%)	1% (0%)	−0.01% (0%)	−3% (−2%)	−0.02% (−0.01%)
UCEC	1% (−1%)	−0.01% (−0.01%)	21% (17%)	0.13% (0.03%)	6% (2%)	0.04% (0%)	3% (0%)	−0.02% (−0.01%)	2% (0%)	0% (0%)	−5% (−5%)	−0.04% (−0.03%)

The added explanatory variances of TFs and lncRNAs are highly correlated between tumour-adjacent and tumour tissue (Fig. 2C), low in miRNA, methylation, and SNPs, and in some cases, adding CNVs to the model decreases explanatory variance (i.e. negative explanatory variance) (Fig. 2C). Pearson correlation analysis between normal and tumour tissue per-regulator shows a high correlation in predictive gain from TFs and lncRNAs, whereas the correlation is low among miRNAs, methylation, and SNPs, and is negative in CNVs (Fig. 2C). The genes best predicted by lncRNAs (e.g. *LEFTY1, C4BPA, HTRA1, AGAP5, DMBT1*, and *RPL11*) are also well predicted by the same models in healthy tissue (Supplementary Fig. S6). We also find that we are able to successfully transfer our cancer models to tumour-adjacent tissue when inspecting the top 6 genes explained by CNVs (i.e. *LLPH, YEATS4, UQCRFS1, POP4, CNOT2*, and *CAND1)* (Supplementary Fig. S7). This is only true of a minority of genes with the majority showing low predictive accuracy from CNVs in tumour-adjacent tissue (Fig. 2B), along with the low correlation of added explanatory value (Fig. 2C), potentially reflecting the high prevalence of aneuploidy in cancers, and not in tumour-adjacent tissue [23, 59]. Alternatively, it’s possible that these results reflect how CNVs are less predictive of gene expression in the context of the other regulators. Previous work has shown a strong correlation between gene copy number and gene expression in TCGA [60]. Our data extends these findings, as CNVs are highly predictive of gene expression if other regulators are not controlled for (Supplementary Fig. S8), but not as predictive when other regulators are present in the model. These results suggest that while the CNV of the gene can impact gene expression, in the majority of cases, the associated copy-number of regulatory elements have the most important impact on gene expression, especially in the context of cancer CNVs. These results are intuitive, as duplicated genes without regulatory architecture are unlikely to be functional and therefore won’t lead to increased expression.

After looking at genes across all cancers, we investigate large-scale regulatory patterns in the TCGA data by clustering predictive model results per cancer. We mark each gene with the top regulator(s) of its cluster (Fig. 2D). We then compare our results across cancers. The expression of genes in most cancers is primarily driven by TFs (12/21 cancers) while the other cancers are primarily driven by DNA methylation (4/21 cancers). Specifically, these four cancers have a squamous histology, namely SARC, BRCA, ESCA, and CESC. Each regulator does not function independently. Accordingly, stomach adenocarcinoma (STAD), bladder adenocarcinoma (BLCA), head and neck squamous carcinoma (HNSC), and liver hepatocellular carcinoma (LIHC) show the largest predictive contribution from TF expression and methylation jointly (Fig. 2A). Overall, TFs and methylation are the top regulators for 83% of the genes in this clustering analysis. The next most common top regulator is miRNA with 8% of the genes showing it as the most predictive, lncRNA is the most predictive for 6%, CNV for 2%, and SNPs for 0.07% of the genes.

The majority of genes show expression dominated by 5 patterns of top regulatory drivers: (i) TFs alone; (ii) methylation alone; (iii) TFs and methylation; (iv) TFs, methylation, and CNVs; and (v) miRNA alone (Fig. 2D). SNPs don’t appear in any of the top regulatory groups, likely because in each cancer, too few genes show a predictive gain directly from *cis-*SNPs and not via other factors in their expression models (Fig. 2A). Groups of genes with a majority of predictive gain from SNPs are therefore small. Nonetheless, we find a subset of genes such as *CRYBB2, RPS26, XRRA1, FAM118A*,and*C2ORF74* (Supplementary Fig. S9), all of which show predictive gains of >50% from SNPs in multiple cancers and tumour-adjacent tissue. We have compiled this compendium of results into iModEst (Integrative Models of Estimated gene expression regulation). Our iModEst atlas stores the explanatory variance of each gene expression regulator in TCGA across >16,000 genes in 21 tissues (sample size for each model: Supplementary Table S1). Users can query these data to gain an understanding of how their genes and gene lists may be regulated regardless of how the genes are previously annotated. Having a deeper understanding of how a set of genes are similarly regulated can be a powerful way to find core genes and regulators in a complex biological process [1, 61]. Overall, we provide an accessible platform for users to explore the most comprehensive integration of multi-omics in the context of explaining gene regulation to date.

### Benchmarking iModEst using COSMIC genes

To assess iModEst on well studied cancer genes we used the tier 1 and tier 2 Catalogue of Somatic Mutations in Cancer (COSMIC) genes (Supplementary Table S2) [62]. Overall, some COSMIC genes such as *PIK3CA*, show that a consistent set of regulators (i.e. TFs and lncRNAs) predict *PIK3CA* expression across cancers. This stable regulation indicates that the cell state is the primary predictive signal in those genes’ expression across multiple cancers (Supplementary Fig. S10). Alternatively, other COSMIC genes like *TP53* are regulated by very different combinations of regulators depending on the cancer, a finding reflected in the literature [63–67]. The variability in regulatory prediction of *TP53* across cancers was unsurprising, as *TP53* has multiple promoters, isoforms, an antisense-transcriptional regulators in *WRAP3*, TF-binding sites of environmentally responsive TFs like NFKb, and context-depending miRNA interactions [68], amongst other regulatory mechanisms (Supplementary Fig. S11) [63–67].

We find genes previously identified as SNP-regulated to have a strong variance-explained of SNPs in HNSC, such as *TP53*(16% added variance explained) and *FES*(11% added variance explained) [69, 70] despite SNPs generally being a poor predictor of gene expression across all genes in these cancers. Likewise, we find CNVs explain a large share of variation for genes in specific cancer types that have previously been linked to CNVs, like KRAS in both LUSC (31% added variance explained) [71–73] and LUAD (33% added variance explained), *MDM2* in LGG (31% added variance explained) [74], *DROSHA* in BLCA (28% added variance explained) [75], *FGFR2* in STAD (26% added variance explained) [76–78], and *EGFR* in LGG (25% added variance explained) [79, 80].

The top COSMIC gene with the most variation explained by miRNA regulation is *NTRK3*, with an average of 71% added variance from miRNA in BRCA. *NTRK3* is known to be highly regulated by multiple miRNA in breast and other tissues [81–83]. The COSMIC gene with the most variation explained by TF coexpression is *EP300*, with an average of 79% added variance explained from TFs in pancreatic adenocarcinoma (PAAD). The top COSMIC gene with the most variation explained by methylation is *NOTCH1*, with an average of 59% added variance explained from methylation in BRCA. This finding is supported by evidence showing methylation-driven regulation of expression for this gene in other studies investigating methylation in breast cancer [84, 85].

### *BCL2* regulation

For every gene, iModEst can be used to predict how individual regulators within each regulator type (e.g. individual methylated promoters, miRNAs, TFs, SNPs, CNVs, and lncRNAs) and combinations of these specific regulators contribute to a given gene’s expression. For example, iModEst can identify that miRNA is strongly associated with gene expression, and that specific miRNA drives that association. We use the B-cell lymphoma 2 (*BCL2*) gene to showcase how iModEst can simultaneously recapitulate well characterized gene regulation [86] of a well-studied gene while predicting previously uncharacterized modes of expression.

**Figure 3.**
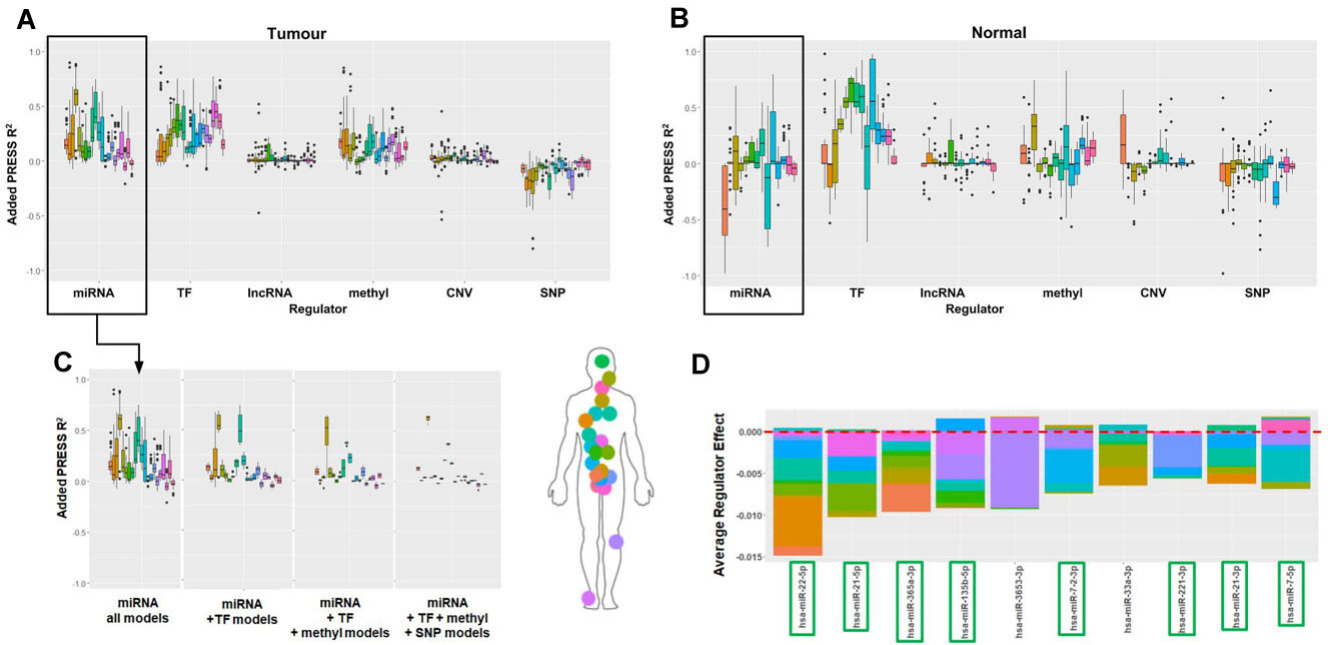
*BCL2* expression regulation dissected. (**A**) Added predictive value per regulator and cancer for *BCL2* expression, showing the highest gains from miRNA, TFs, and methylation. (**B**) Predictive gains from miRNA persist in ESCA, KIRP, LUAD, and PAAD when the same model is applied to normal, tumour-adjacent tissues. (**C**) The gain in predictive accuracy from miRNA decreases when considering all models, as in panel (A), and comparing it to only models that account for TFs, TFs + methylation, and TFs + methylation + SNPs. (**D**) MiRNA with largest negative effects on *BCL2* expression across multiple cancers. MiRNA outlined in green have been shown to functionally target *BCL2*.

Our models showed that miRNAs and TFs are the greatest contributors to the explanation of *BCL2* gene expression variation, conferring a predictive gain of 15–60% in 9 of our 21 cancers for miRNA and <25% in 11 of our 21 cancers for TFs (Fig. 3). Accordingly, previous studies have found that *BCL2* is subjected to strong miRNA and TF regulation *in vitro*, in tumour samples, and in animal models [87–91]. For example, 8 of the top 10 miRNAs most strongly associated with *BCL2* expression across all cancers in iModEst have been shown to functionally regulate the *BCL2* gene in previous literature (Fig. 3D) [87–91]. Therefore, previously identified regulatory factors, such as miRNA targeting *BCL2* expression, are highly complex and tissue-specific.

Our conditional models also show that miRNAs and TFs do not function independently of one another, which to our knowledge has not yet been investigated in the context of *BCL2*. Conditioning our models on TFs, we see the gains in prediction from miRNAs drop in all cancers except CESC, ESCA, and LUAD. Furthermore, when we add methylation to the models of *BCL2*, the added variance of miRNA was increased by >25% in LUAD and ESCA, suggesting that the role of miRNA may be conditional on *cis-*methylation in these cancers. Finally, when only looking at models with SNPs + methylation + TFs included, we found the predictive gain from miRNA in esophageal cancer strengthening, suggesting the effect of miRNA on *BCL2* expression in esophageal cancer is conditioned on *BCL2*’s *cis-*SNPs. These analyses point to TFs and miRNA regulating *BCL2* while likely interacting with one another.

In addition to retrieving the general modalities of regulation, iModEst contains information on specific regulators. We found that, of the top 10 miRNAs identified by our models, 8 miRNA have been shown to regulate *BCL2* expression in prior studies (miR-22-5p [92–94], miR-21-5p [95–100], miR-365a-3p [88, 101], miR-135b-5p [102], miR-7-2-3p [103, 104], miR-221-3p [94], miR-21-3p [95–100], and miR-7-5p [103, 104]), one targets *p53* which targets *BCL2* (miR-33a-3p [86, 105, 106]), and one (miR-3653-3p) has very little available information except that its expression has been observed to increase while *BCL2* expression decreases when comparing cervical cancer to tumour-adjacent tissue [107] (Fig. 3). The effects of individual regulators in high-accuracy predictive models are of great interest when trying to contextualize functional experiments. Therefore, our atlas provides breadth, as well as depth, of per-gene regulation across tissues.

### *CRYBB2* regulation

**Figure 4.**
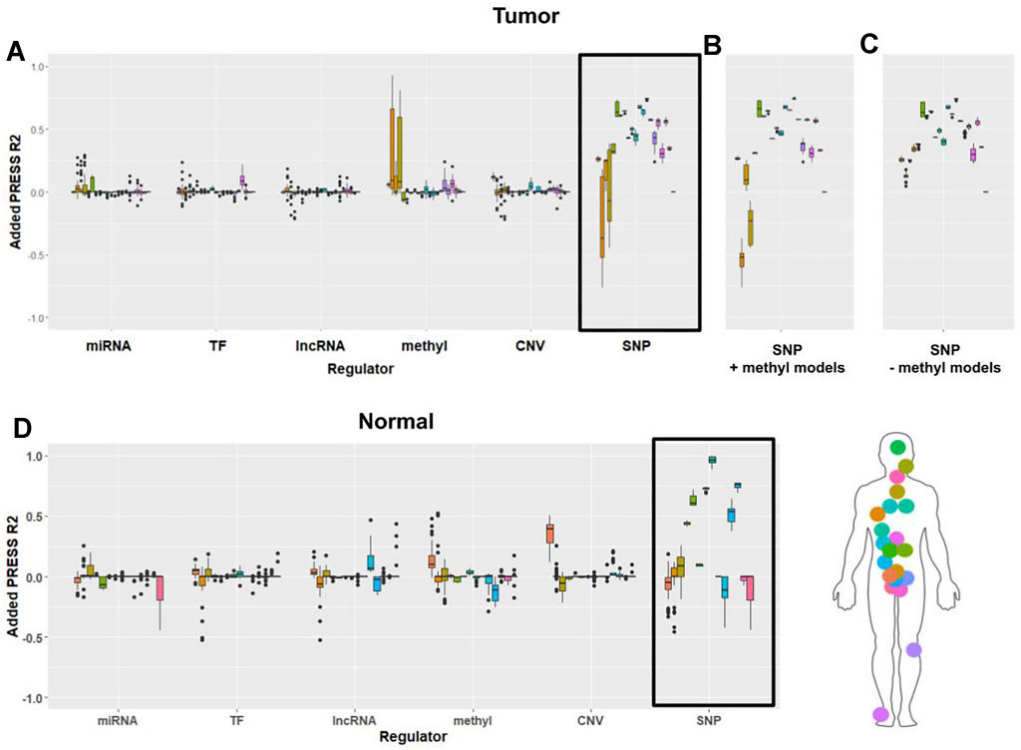
Decomposing the predictive accuracy of SNPs for *CRYBB2* expression. (**A**) Predictive accuracy gains across six regulators over all models for *CRYBB2*. SNPs are highly and almost independently predictive of *CRYBB2* gene expression except in the cases of two cancers: BRCA and ESCA. (**B**) Predictive accuracy gains from SNPs ±1 MB of *CRYBB2’s* gene boundary when added to models already including methylation. Here, we see a drop in SNP predictive accuracy for BRCA and ESCA when only models including methylation are considered (**C**) Predictive accuracy gains from SNPs when added to models with no methylation data included. The gain in predictive accuracy from SNPs for BRCA and ESCA jumps to a positive value when added to models with methylation excluded while it remains stable and largely unchanged for other cancers. (**D**) Gain in predictive accuracy from each regulator in normal samples (using models fit with tumour samples). Here, we see the gain in predictive accuracy from SNPs is maintained in the majority of tissues.

iModEst provides an unbiased characterization of which regulators contribute to gene expression, providing an unprecedented opportunity to investigate the regulation of genes that are not as well characterized as genes like *TP53* and *BCL2* [22]. We chose to showcase *CRYBB2* because it demonstrates a different pattern of regulation from *BCL2* and because of the role of SNPs in predicting its expression, which was uncharacteristic of most genes in iModEst. SNPs, which include common imputed variants within 1MB of the gene of interest in our models, were found to explain >25% variance for this gene in 15 tumour tissues and 6 tumour-adjacent tissues (Fig. 4) [108]. For this reason, we sought to further characterize the degree to which each regulator impacts prediction of this gene’s expression and dissect how these regulators interact. For BRCA and ESCA, our findings suggest the *cis-*eQTL effect is mediated by methylation. We are able to further investigate this mediation by comparing the predictive gains from SNPs when added to models with methylation included and excluded. We see that when methylation is included in the predictive model for *CRYBB2* expression, adding SNPs no longer leads to a gain in predictive accuracy in BRCA and ESCA (Fig. 4B). In contrast, SNPs added to models in which methylation is excluded show a 25% median gain in variance explained in these same cancers (Fig. 4C), suggesting that the variation explained by SNPs is also explained by methylation. Cases of shared variation between regulators are easily spotted in iModEst by observing very large boxplot inter-quartile ranges, indicating a subset of models in which the regulator adds a great deal to the variation explained and a subset of models when the same regulator contributes much less.

We further investigated which specific variants were driving the effect of SNPs on gene expression in LGG, a cancer where SNP prediction was high and independent of other regulatory factors (Fig. 4). Overall we found 47 SNPs which had variation associated with gene expression (i.e. elastic net coefficient > 0) of *CRYBB2* in LGG (Supplementary Fig. S12A), with these SNPs clustering at the 5′ and 3′ end of the primary transcript (i.e. elastic net coefficient > 0). We also identified (Supplementary Fig. S12B) a cluster of methylated CpGs at the 5′ end of the transcript with variation associated with gene expression. The most predictive SNPs, rs374222410, were within 1 kb of the 3′ end of *CRYBB2* and did not show evidence of TF, miRNA, or DNA methylation interactions (Supplementary Fig. S12B). As such the predictive impact of SNPs on *CRYBB2* in *LGG* is likely associated with an additive effect of 5′ SNP variation being mediated by DNA methylation, and 3′ variation possibly being mediated by regulatory modalities not captured in this study (e.g. variable repeat length in rs374222410 influencing DNA topology).

### Interacting with iModEst

iModEst tests the dependence of multiple regulatory modalities by conditioning the baseline models on a particular regulator, and evaluating whether the predictive accuracy of the other regulators is altered. Users interacting with iModEst can perform these analyses themselves. This web application also has our iModEst data for download so users can replicate all figures of this analysis as well as explore further in any fashion with the explained variance and coefficient data for each gene and cancer. In addition, we visualized SNP and methylation coefficients using the UCSC genome browser instead of the web application since this allows users to layer other location-specific information at the given genomic coordinates. Specifically, we grouped the weighted coefficients for every gene–SNP or gene–CpG pair and visualized the coefficients with a non-zero effect size (Supplementary Fig. S12B). We also measured the magnitude and the sum of each SNP or CpG and converted them into bigwig files, allowing users to visualize the effect size of each SNP or CpG along the genome.

## Discussion

Here, we present iModEst, which integrates multi-omic data from TCGA to predict how six gene regulatory mechanisms (miRNA, TFs, lncRNA, methylation, CNVs, and SNPs) influence gene expression of over 16,000 genes across 21 cancers. iModEst uses a computationally intensive approach to estimate the shared and independent impacts of these regulators on gene expression. This enables iModEst to define a new baseline for the relationship between many genes and regulators, some of which have not been studied in-depth up to this point. By using a well-studied data set with established pre-processing, this work adds a new dimension to the many pan-cancer investigations of TCGA. In so doing, findings from iModEst can be validated based on independent analyses of the same data as well as studies in independent data sets.

iModEst identifies patterns of genomic regulation, some of which expected while others are surprising. For example, we find that TF expression is the most predictive regulator, as expected [109], and *cis-*methylation is the dominant predictive regulator type in cancer sets containing squamous tissue [5, 9] (Fig. 2). In contrast to these strong regulatory predictors of gene expression, we find extremely localized predictive accuracy from our overall least predictive regulator: SNPs. This led us to another expected finding, namely that *cis-*SNPs act as a mediator to other regulators’ influence on gene expression, as we demonstrate with *BCL2* and *CRYBB2*. Indeed, a major benefit of this investigation is our ability to decompose the predictive gain that is shared versus complementary between multiple regulators. We pass this ability on to our readers by providing our raw results as well as a web application which enables users to explore our findings across regulators as well as along the genome in the UCSC genome browser [110].

While we show many expected global and localized patterns in genomic regulation, iModEst contains a trove of new and interesting relationships. We note a few of these relationships in this work, such as the overwhelming predictability of *CRYBB2* expression using *cis-*SNPs alone, however, we leave innumerable other patterns and relationships for users to discover on their own.

### Limitations

First, iModEst does not predict the biological underpinnings of all the regulatory associations found. For example, a gene may be regulated by a set of TFs due to tissue and cell-type specificity, due to cancer-specific gene expression, due to the baseline regulation of that gene, or by a combination of these factors. In this study, we investigated the role that tumour-specific regulation has on gene expression predictions and tissue-specific expression may have on gene expression predictions. In addition, our desire to maximize our sample sizes provides two limitations to iModEst. First, we needed assays that measure regulators in many samples. For example, we use gene expression of TFs to model TF regulation on the genes which have been previously shown to bind in any tissue/cell-type in ENCODE [16]. Second, to allow for the maximum sample size, major cancers were grouped together and histological subtypes were corrected for rather than separating samples into each subtype [5, 6, 9]. Next, this analysis focuses on the TCGA cancer data and its generalization to non-cancer, tumour-adjacent tissue; however, we believe an analysis of this kind in non-pathologic data will likely be fruitful and deliver distinct results, particularly in terms of epigenetic regulation which is highly altered in the tumour microenvironment. As further data are collected and large-scale assays are performed [111–113], tools such as iModEst, and the analytical techniques it employs, can be extended to explain even more variance in gene expression across contexts.

The TCGA data used to build iModEst lack single-cell resolution, making it challenging to disentangle gene expression patterns arising from cell-type composition rather than regulatory state. We found *cis-*SNPs to be limited in their predictive power. This is due to the agnostic inclusion of all imputed, common SNPs within 1 Mb of the gene, removing many rare cancer hotspot markers and somatic variations. We believe this to be the best approach as this has allowed us to still identify several genes (such as *CRYBB2*, *RPS26*, and *XRRA1*) which appear to be highly regulated by *cis-*SNPs. However, stricter inclusions of SNPs (e.g. to previously identified eQTLs) likely would have resulted in a larger predictive contribution from this regulatory group.

Last, in our effort to include many data types, we directly use preprocessed gene-level CNV values from TCGA as it allows us to include CNVs in our elastic net regression as predictors, similar to the other regulators included. However, in contrast to the many SNPs included as predictors, CNVs have only 1 predictor value for each gene using this method, thus, CNVs tend to show a low predictive contribution in the elastic net models, which contrasts a previously discovered moderate correlation between gene-level CNVs and gene expression discovered within different cancers and cell-types. Nonetheless, this work identifies genes such as *LLP4*, *POP4*, and *UQCRFS1*, which show very high regulatory control by CNVs. Therefore, in spite of this conservative approach, genes which are highly regulated by CNV are still identified in a cancer-specific manner.

## Conclusion

We have created iModEst as a reference atlas for researchers to gain a global and local insight into the regulatory mechanisms involved in gene expression across the genome. iModEst replicates findings of well-known genes and predicts reasonable regulatory control of unknown genes, generating potential targets for future experimentation. The methodological innovations in developing the iModEst model provide a powerful groundwork for modeling gene expression in future datasets. As scientists uncover novel gene-regulatory associations across conditions and diseases, iModEst will be aresource for interpreting new discoveries and facilitating hypothesis generation toward how these discoveries fit into larger regulatory contexts.

## Supplementary Material

lqaf011_Supplemental_Files

## Data Availability

Data for this project is available from the Cancer Genome Atlas (https://portal.gdc.cancer.gov/). Our data visualization tool, as well as all data produced by this work, including a complete list of sample IDs and regulators used in every gene model can be found at https://imodesttool.org/, with variation of gene expression explained (PRESS R2) additionally shared at https://zenodo.org/records/14649110. Methylation UCSC track hub data can be found at http://wilsonlab.org/public/iModEst/methyl_hub/hub.txt. SNP UCSC track hub data can be found at http://wilsonlab.org/public/iModEst/SNP_hub/hub.txt.

## Code availability

Code for this project is available from https://github.com/larunerdman/imodest/, describing data and development of the iModEst model and enabling the replication of this project’s figures and tables based on model outputs. Models can also be explored and figures replicated using our data visualization tool at https://imodesttool.org/ where our iModEst model output can also be downloaded and explored by users.
