# Supplementary material for: iModEst: disentangling -omic impacts on gene expression variation across genes and tissues
Source: NAR Genom Bioinform. 2025 Mar 4;7(1):lqaf011. doi: 10.1093/nargab/lqaf011 (PMC11879402; doi:10.1093/nargab/lqaf011)
Supplement: lqaf011_Supplemental_Files [file lqaf011_supplemental_files.zip › Modes_SuppFigures_20250117.docx]

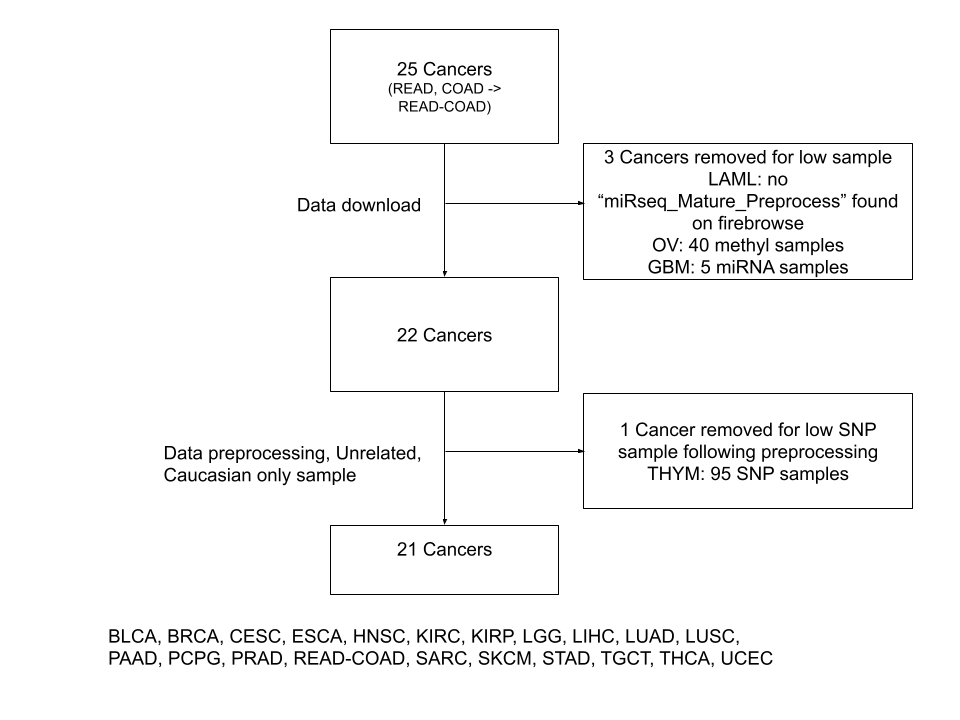


**Supplementary Figure 1.** Consort diagram of all cancers included in analyses.


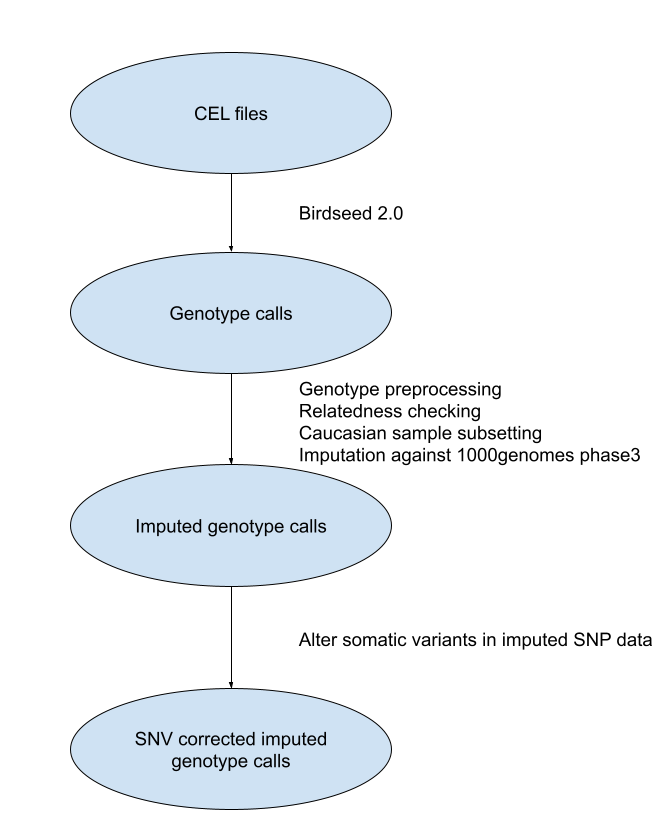


**Supplementary Figure 2.** SNP preprocessing pipeline from Affymetrix intensity files to common variant genotype calls.


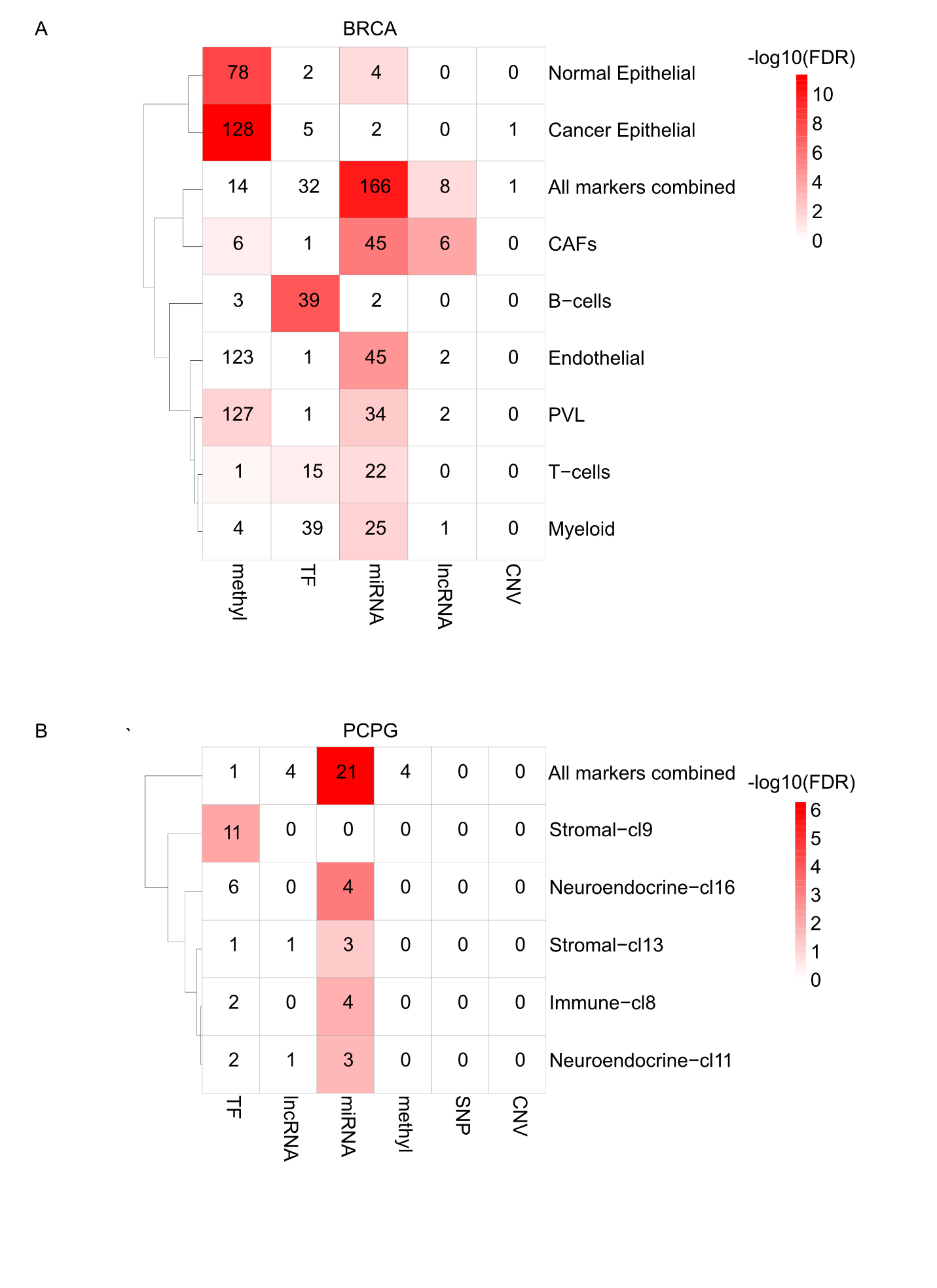


**Supplementary Figure 3.** Enrichment of genes predicted by each regulator (>25% predictive gain) against cell-type markers from public snRNA-seq studies investigating (A) BRCA and (B) PCPG. Heat in the heatmaps represents the -log10(FDR-adjusted p-value) from an unranked hypergeometric test, and the heatmaps are populated by the number of genes that are cell-type markers (rows) and predicted by each regulator (columns).


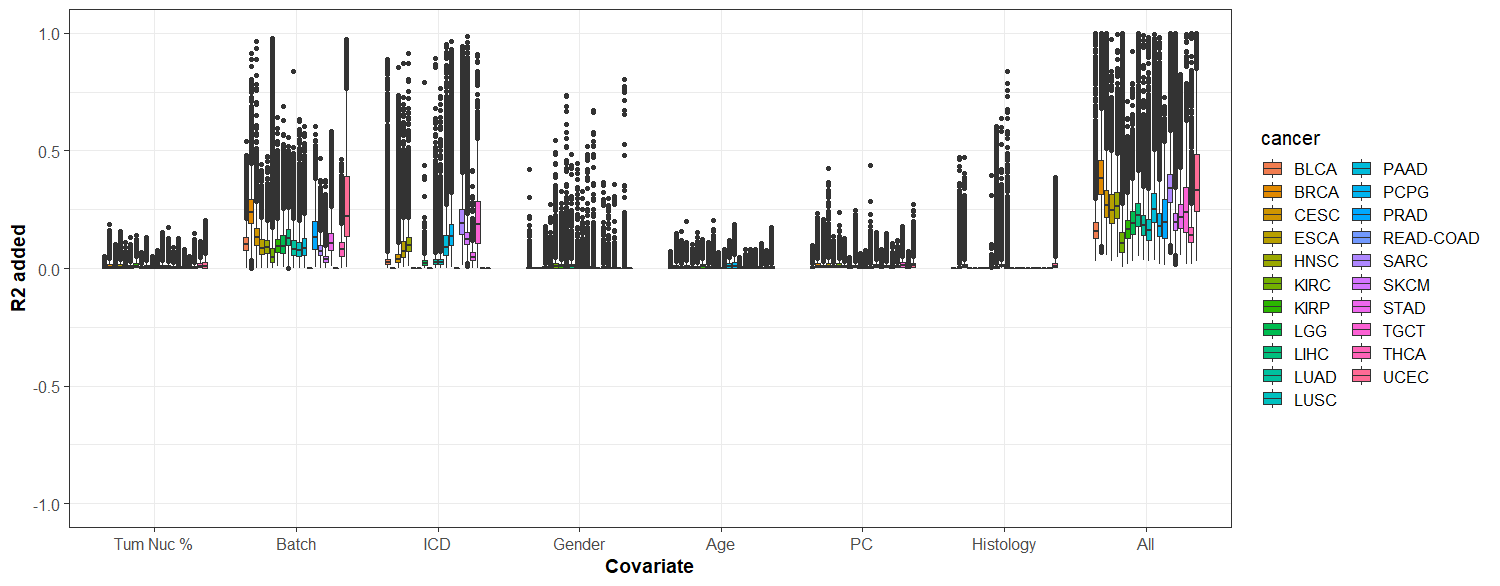


**Supplementary Figure 4.** Added variability of gene expression explained for each gene in each cancer for each covariate/confounder category.


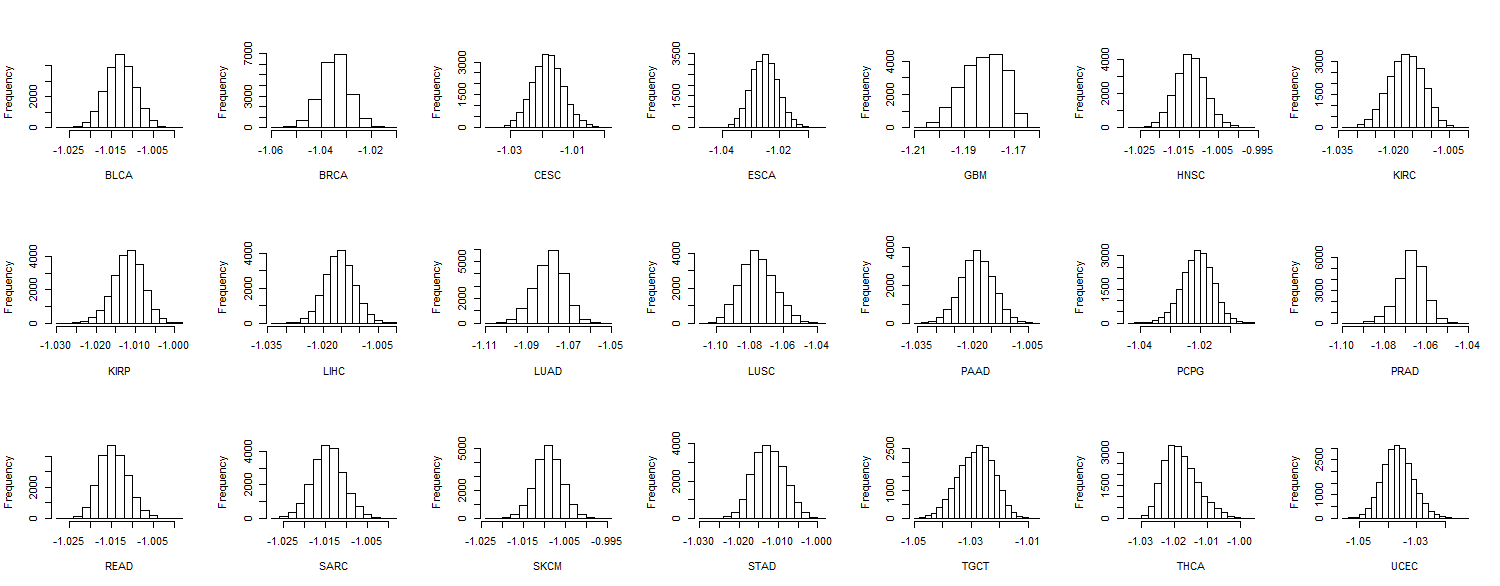


**Supplementary Figure 5.** Deflation statistics for genes in each cancer. All deflation statistics are below 0, therefore PRESS R^2^ values above 0 are not occurring in our analyses by random chance.


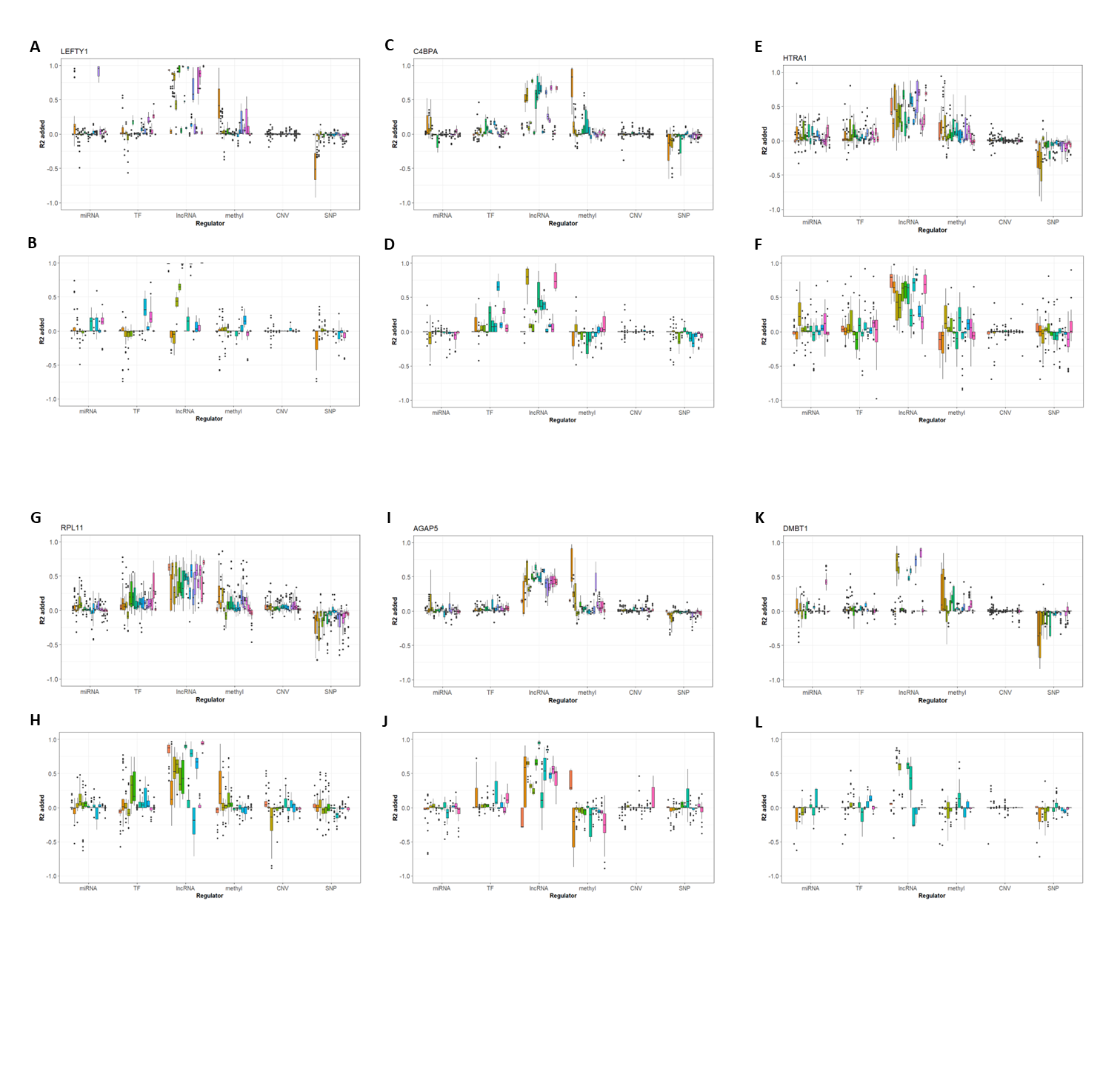


**Supplementary Figure 6.** PRESS R^2^ added in top 6 genes predicted by lncRNA (A) *LEFTY1* in tumour data, (B) *LEFTY1* in normal, tumour-adjacent tissue, (C) *C4BPA* tumour data, (D) *C4BPA* normal, tumour-adjacent tissue, (E) *HTRA1* in tumour data, (F) *HTRA1* in normal, tumour-adjacent data, (G) *RPL11* in tumour data, (H) *RPL11* prediction in normal, tumour-adjacent data, (I) *AGAP5* in tumour data, (J) *AGAP5* in normal, tumour-adjacent data, (K) *DMBT1* prediction in tumour data, (L) *DMBT1* prediction in normal, tumour-adjacent data.


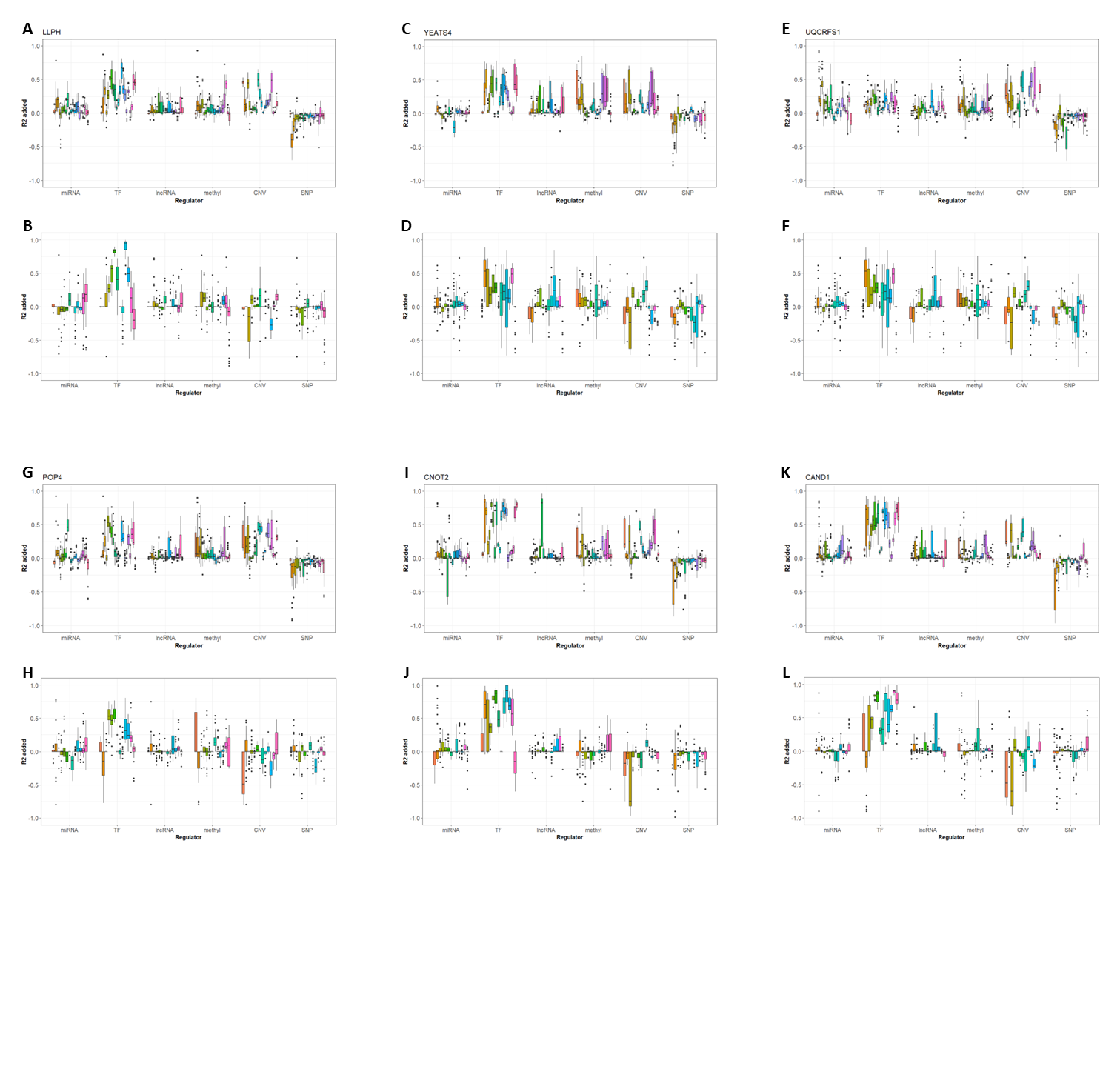


**Supplementary Figure 7.** PRESS R^2^ added in top 6 genes predicted by CNVs (A) *LLPH* in tumour data, (B) *LLPH* in normal, tumour-adjacent tissue, (C) *YEATS4* tumour data, (D) *YEATS4* normal, tumour-adjacent tissue, (E) *UQCRFS1* in tumour data, (F) *UQCRFS1* in normal, tumour-adjacent data, (G) *POP4* in tumour data, (H) *POP4* prediction in normal, tumour-adjacent data, (I) *CNOT2* in tumour data, (J) *CNOT2* in normal, tumour-adjacent data, (K) *CAND1* prediction in tumour data, (L) *CAND1* prediction in normal, tumour-adjacent data.


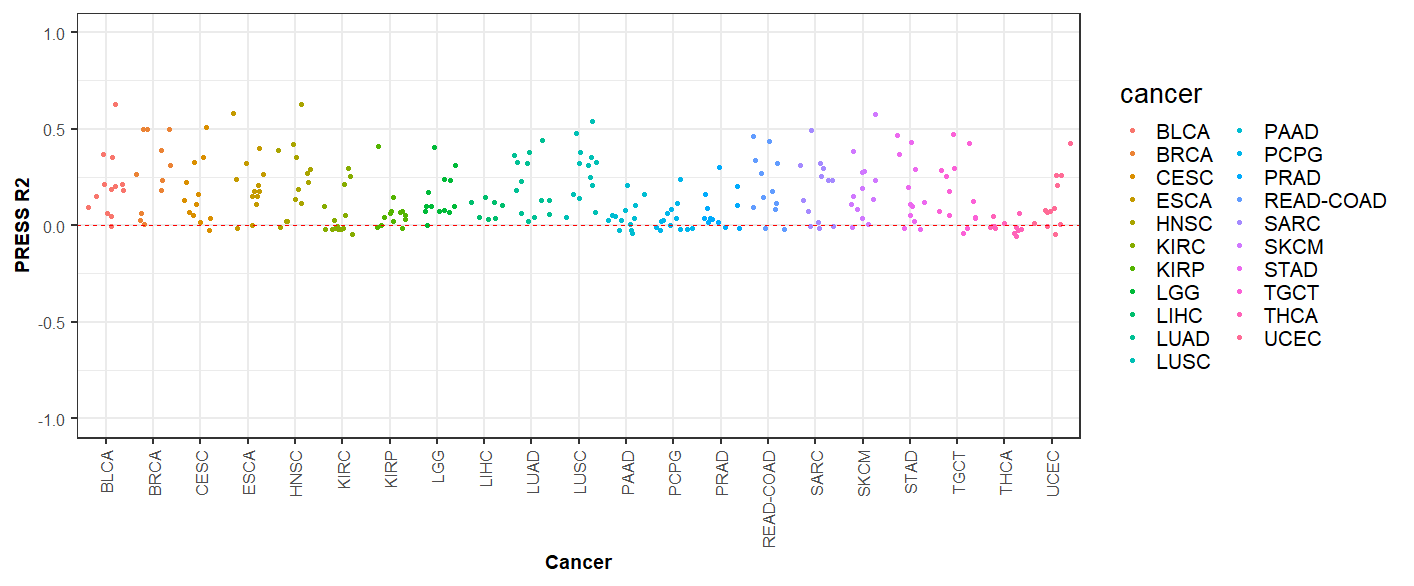


**Supplementary Figure 8.** Marginal PRESS R^2^ statistics for CNV along in the following genes in Shao et al, 2019: *MYC, AKT1, CDK9, KRAS, MDM2, CDKN2A, RB1, IDH2, NPM1, PRAME, MYCN, FAM60A, FGFR1, STK11* (<https://doi.org/10.1186/s12881-019-0909-5>).


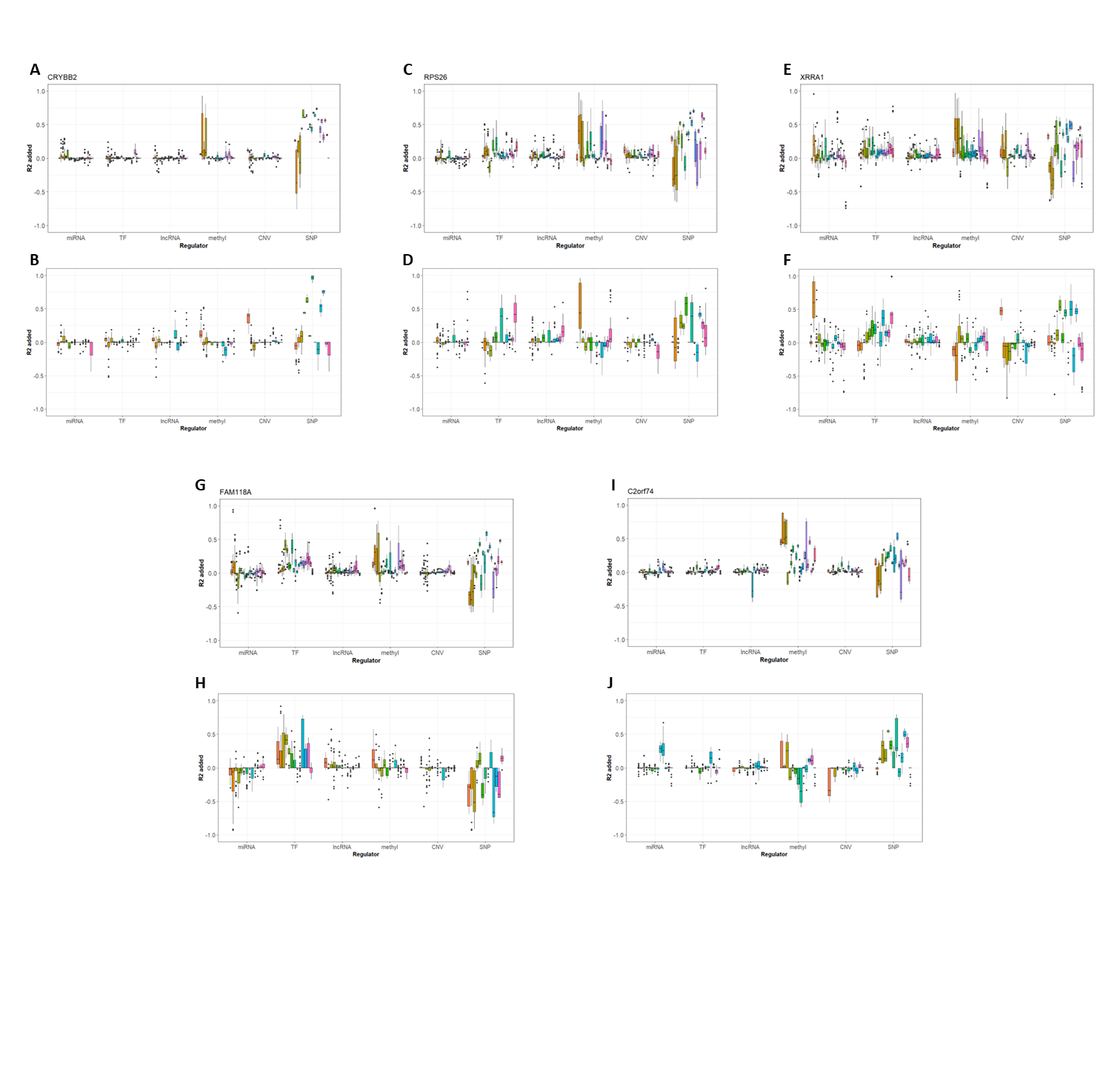


**Supplementary Figure 9.** PRESS R^2^ added in top 5 genes predicted by SNPs (A) *CRYBB2* in tumour data, (B) *CRYBB2* in normal, tumour-adjacent tissue, (C) *RPS26* tumour data, (D) *RPS26* normal, tumour-adjacent tissue, (E) *XRRA1* in tumour data, (F) *XRRA1* in normal, tumour-adjacent data, (G) *FAM118A* in tumour data, (H) *FAM118A* prediction in normal, tumour-adjacent data, (I) *C2orf74* in tumour data, (J) *C2orf74* in normal, tumour-adjacent data.


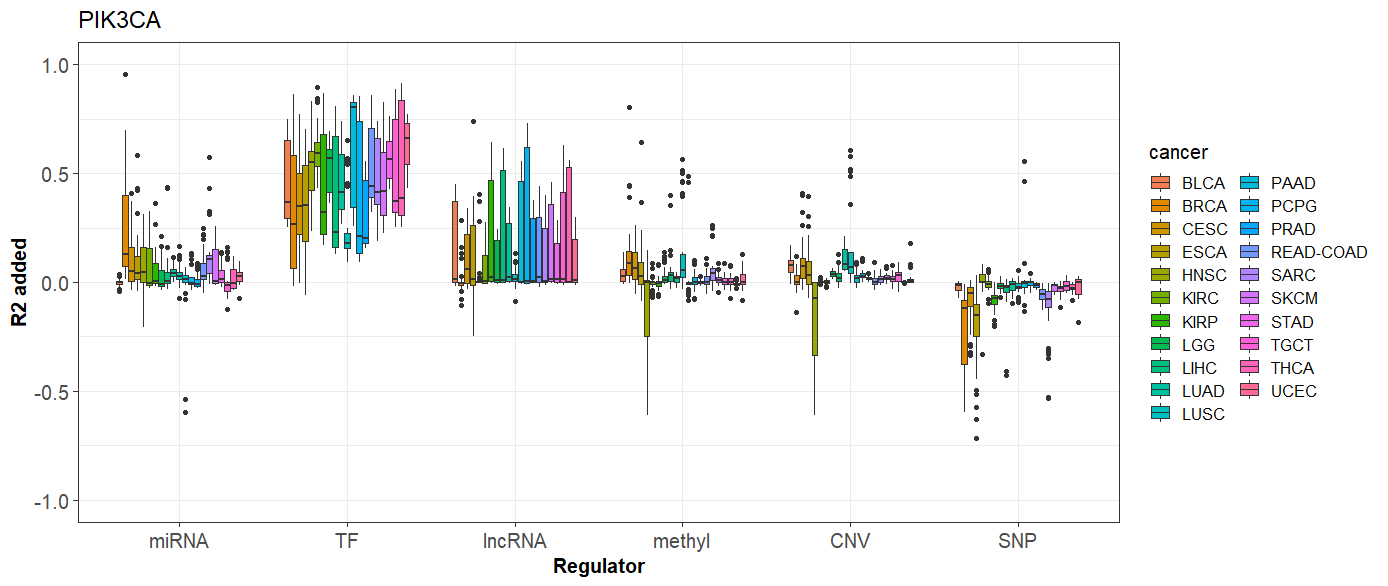


**Supplementary Figure 10.** PRESS R2 for *PIK3CA* in tumour tissue.


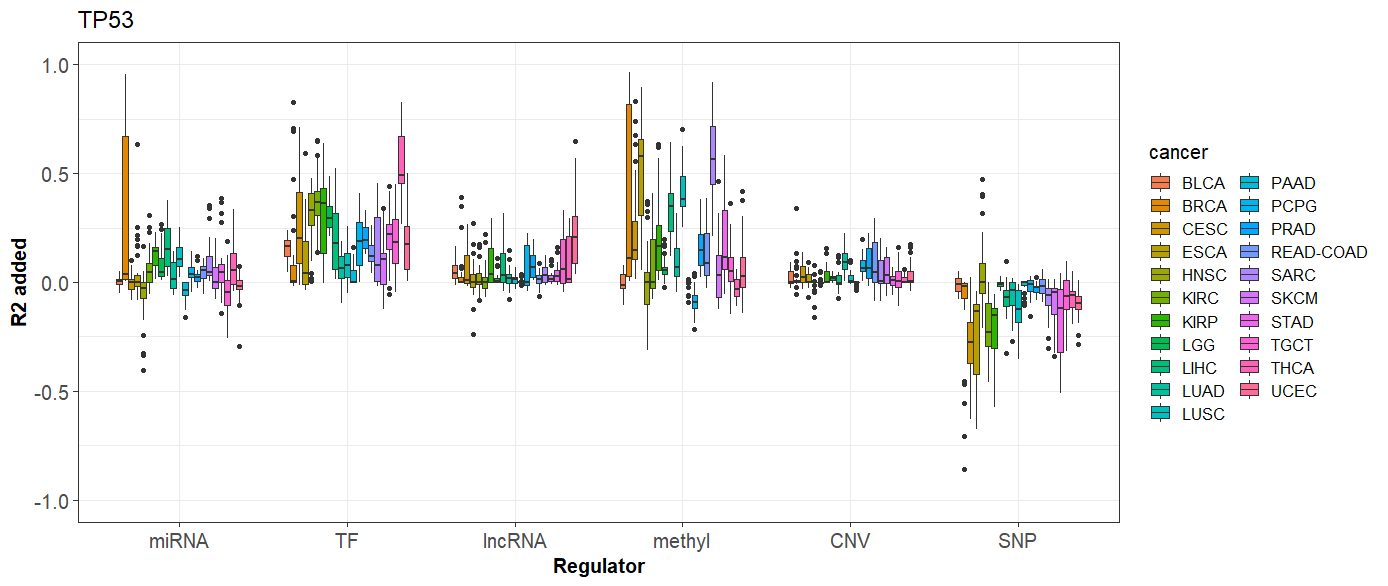


**Supplementary Figure 11.** PRESS R2 for *TP53* in tumour tissue.

**
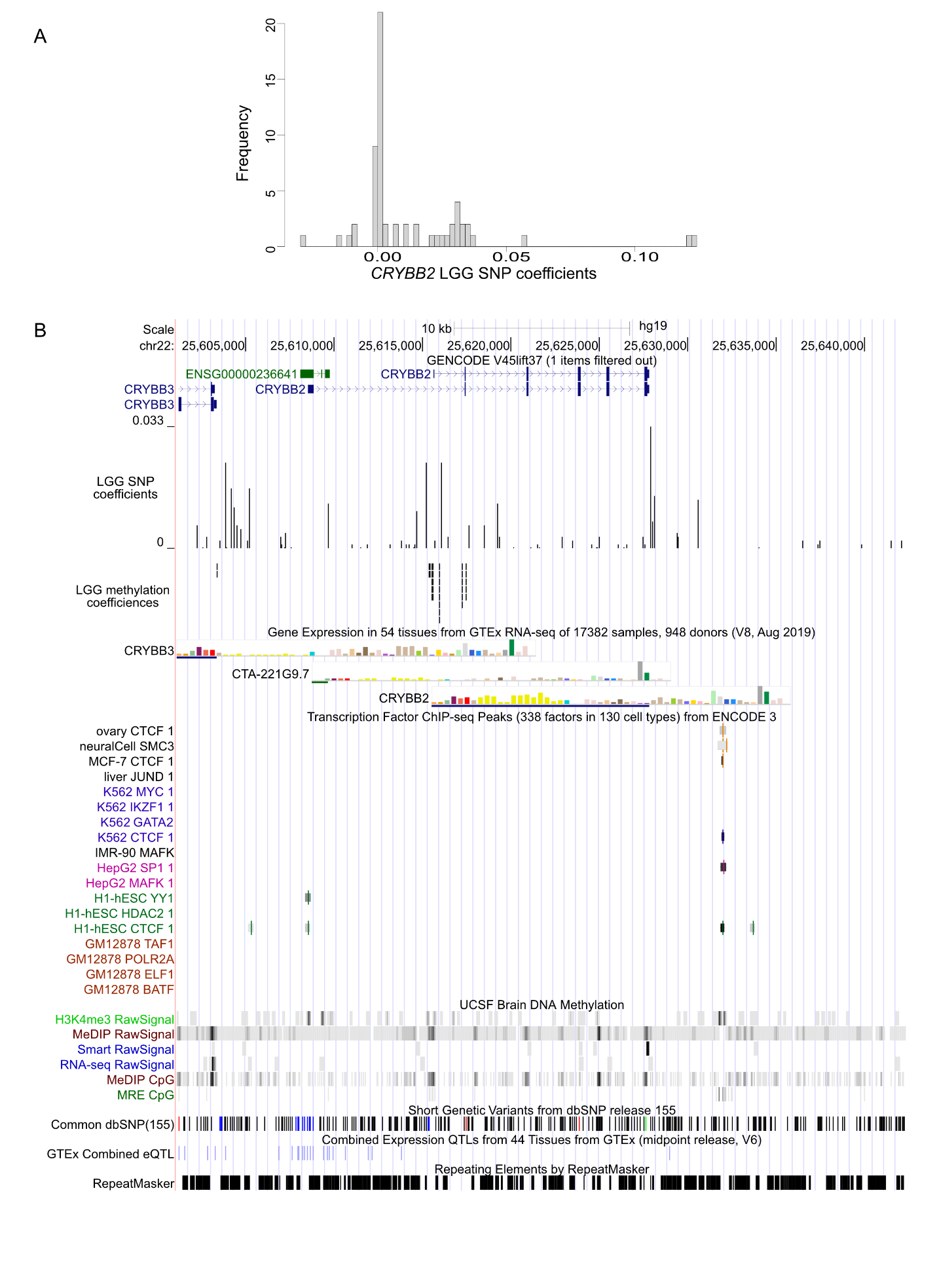
**

**Supplementary Figure 12.** Locus-level summary of SNPs and DNA methylation predictions of the *CRYBB2* gene in lower grade glioma (LGG). A) Histogram displaying the coefficients of the 47 SNPs that have a non-zero predictive gain of *CRYBB2* in LGG. B) Genome browser screenshot of *CRYBB2* using the UCSC browser and a series of browser tracks that relate to *CRYBB2* regulation. From top to bottom, these browser tracks are: gene models, histogram of SNP coefficients from iModEst, histogram of DNA methylation coefficients from iModEst, *CRYBB2* gene expression in the genotype tissue expression consortium (GTEx) database, condensed histogram of transcription factor binding sites from ENCODE3, condensed epigenetic and gene expression signal from the UCSF database, SNPs detected in dbSNP, expression quantitative trait loci (eQTL) identified in GTEx, and lastly condensed annotations of repeat regions.
