# Supplementary material for: iModEst: disentangling -omic impacts on gene expression variation across genes and tissues
Source: NAR Genom Bioinform. 2025 Mar 4;7(1):lqaf011. doi: 10.1093/nargab/lqaf011 (PMC11879402; doi:10.1093/nargab/lqaf011)
Supplement: lqaf011_Supplemental_Files [file lqaf011_supplemental_files.zip › SuppFile1_ iModEst 1000Genomes PCA plots.docx]

This document shows the PCA plots and PC boundaries chosen to identify population outliers. The colors in the graph breakdown in the following way:

Pink = Our data

Red = Identified outliers

Cyan = Tuscan (TSI)

Black = Iberian (IBS)

Blue = Caucasian Europeans from Utah (CEU)

Green = British (GBR)

Orange = Finish (FIN)

**BLCA**

**
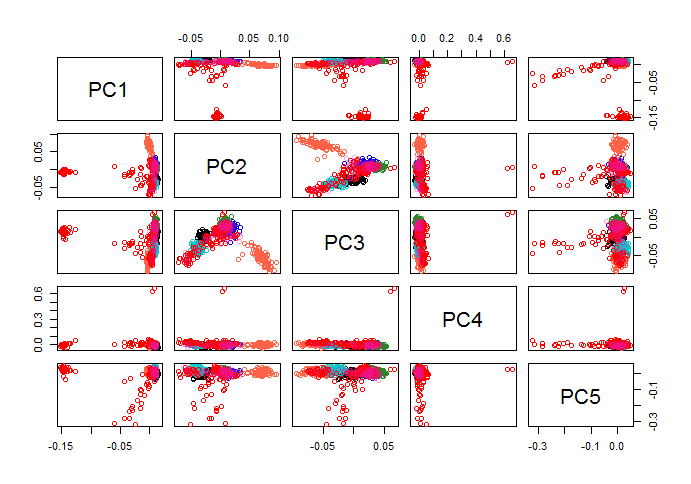
**

**
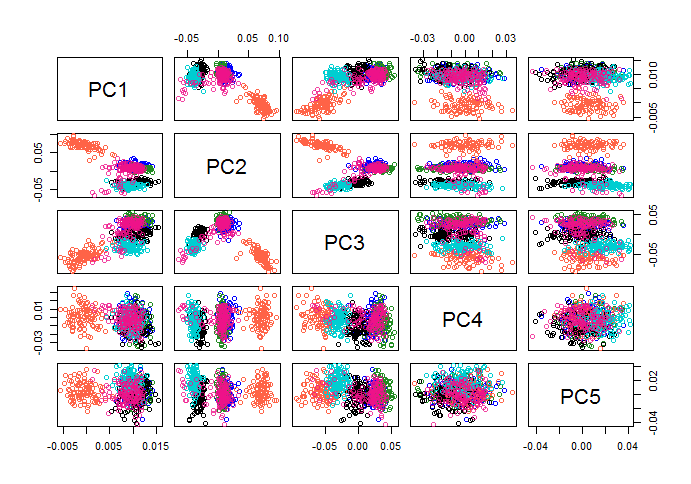
**

eigenvector$pop1kg[eigenvector$PC1 < -0.01] <- "ol"

eigenvector$pop1kg[eigenvector$PC4 > 0.015] <- "ol"

eigenvector$pop1kg[eigenvector$PC5 < -0.05] <- "ol"

**BRCA**


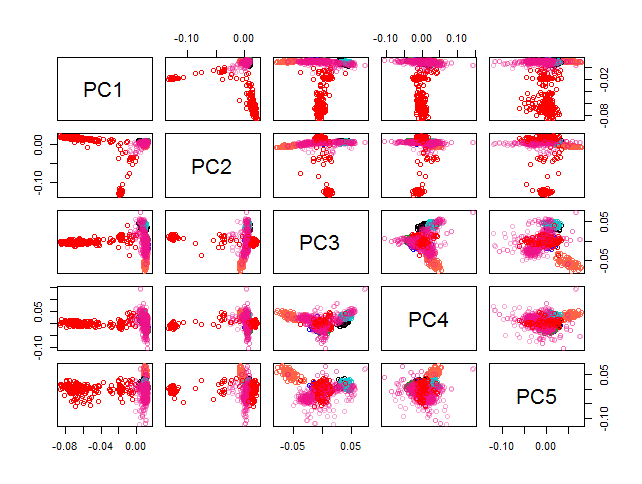


eigenvector$pop1kg[eigenvector$PC1 < -0.01] <- "ol"

eigenvector$pop1kg[eigenvector$PC2 < -0.03] <- "ol"

**CESC**

**
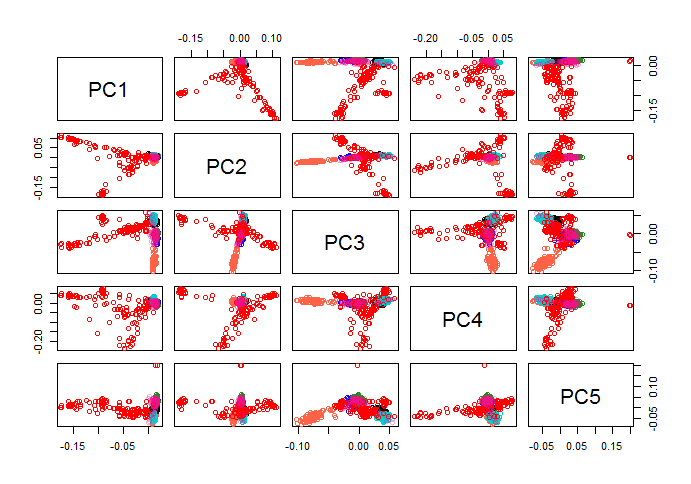
**

**
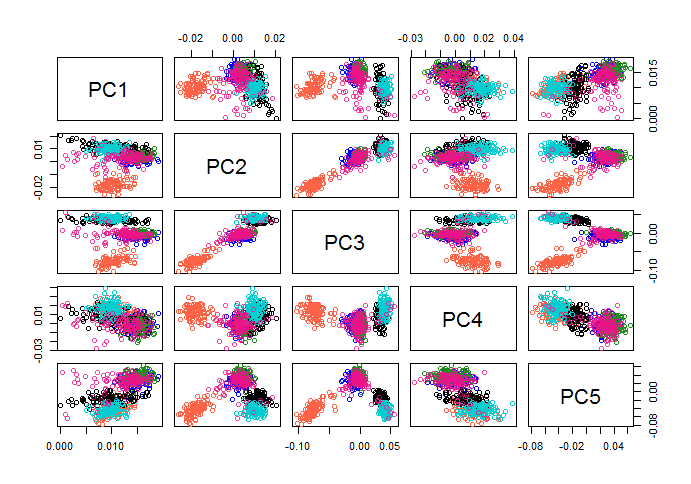
**

eigenvector$pop1kg[eigenvector$PC1 < -0.001] <- "ol"

eigenvector$pop1kg[eigenvector$PC5 > 0.1] <- "ol"

eigenvector$pop1kg[eigenvector$PC4 < -0.03] <- "ol"

**COAD**

**
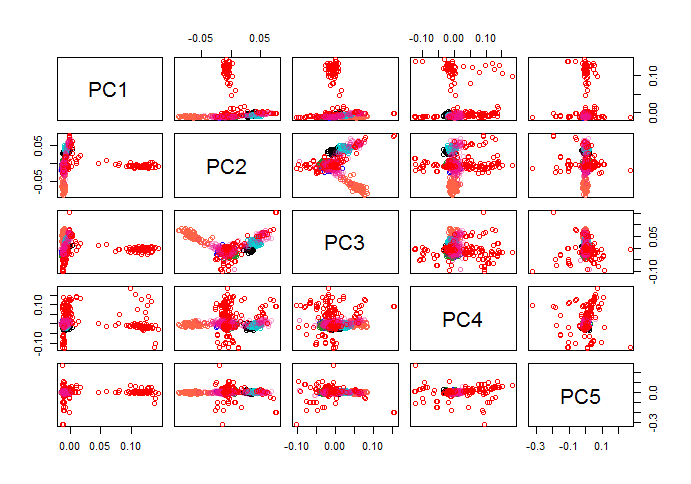
**

**
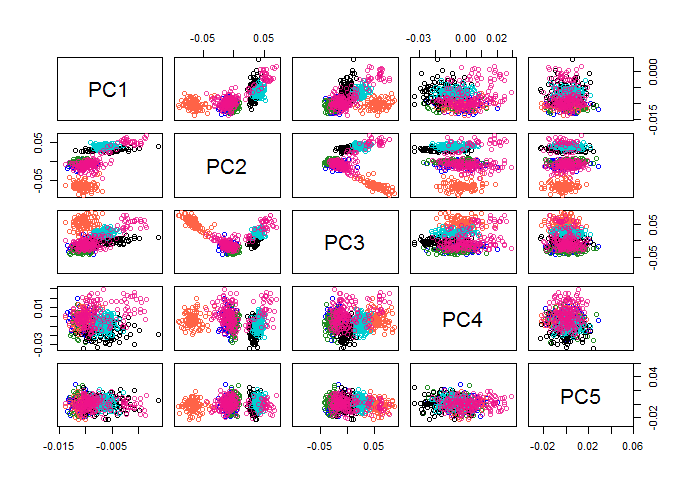
**

eigenvector$pop1kg[eigenvector$PC1 > 0.004] <- "ol"

eigenvector$pop1kg[eigenvector$PC5 > 0.07] <- "ol"

eigenvector$pop1kg[eigenvector$PC4 > 0.03] <- "ol"

eigenvector$pop1kg[eigenvector$PC5 < -0.035] <- "ol"

eigenvector$pop1kg[eigenvector$PC4 < -0.05] <- "ol"

**ESCA**

**
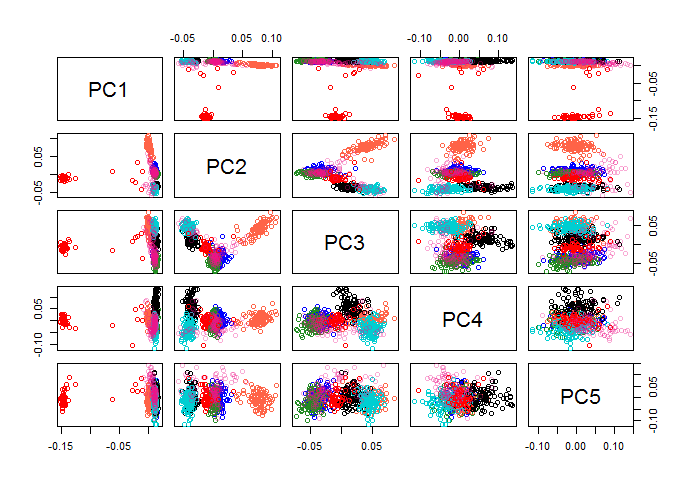
**

**
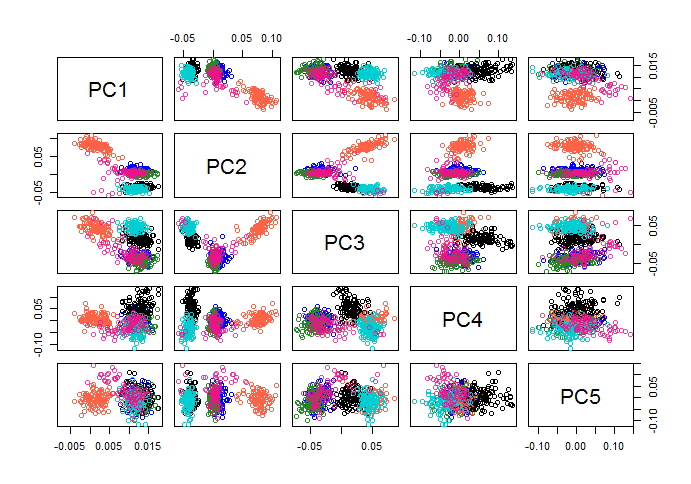
**

eigenvector$pop1kg[eigenvector$PC1 < -0.008] <- "ol"

**HNSC**

**
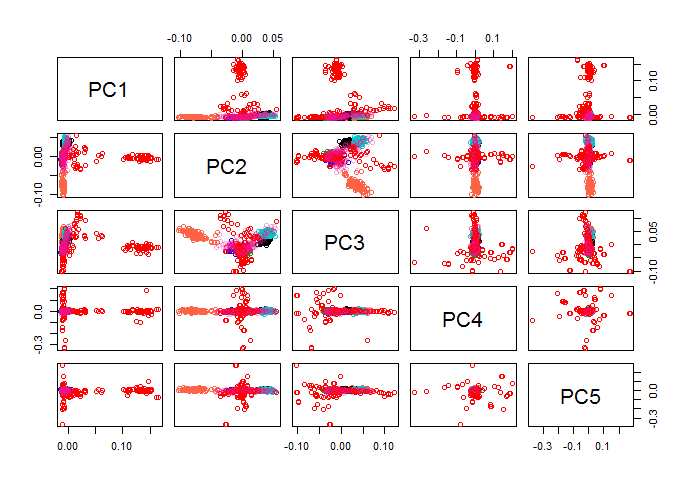
**

**
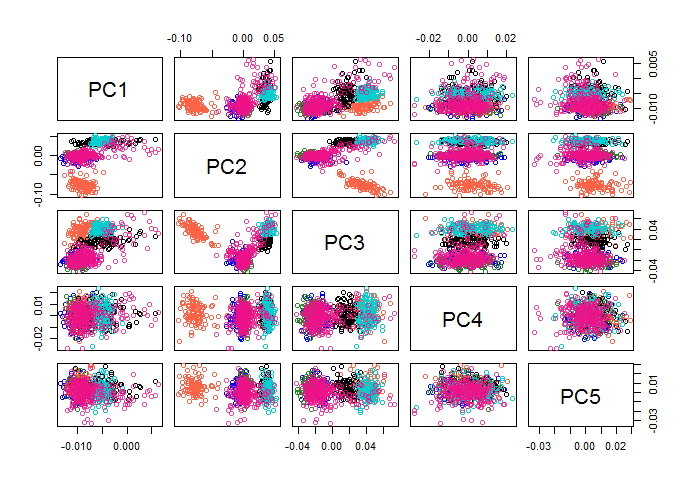
**

eigenvector$pop1kg[eigenvector$PC1 > 0.007] <- "ol"

eigenvector$pop1kg[eigenvector$PC5 > 0.03] <- "ol"

eigenvector$pop1kg[eigenvector$PC4 > 0.03] <- "ol"

eigenvector$pop1kg[eigenvector$PC5 < -0.035] <- "ol"

eigenvector$pop1kg[eigenvector$PC4 < -0.035] <- "ol"

eigenvector$pop1kg[eigenvector$PC3 < -0.06] <- "ol"

**KIRC**

**
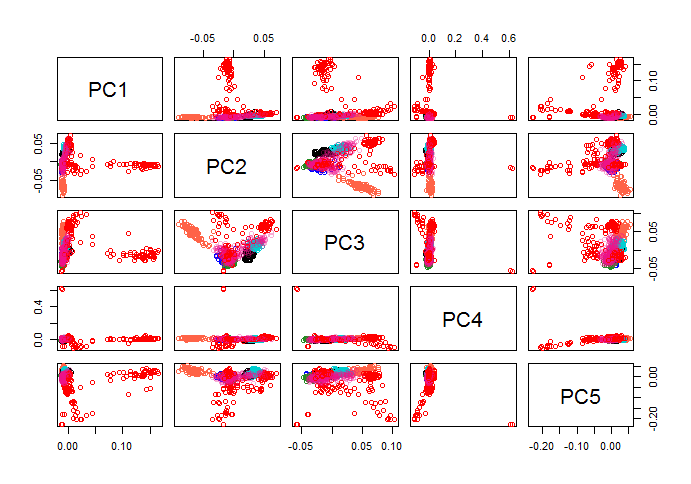
**

**
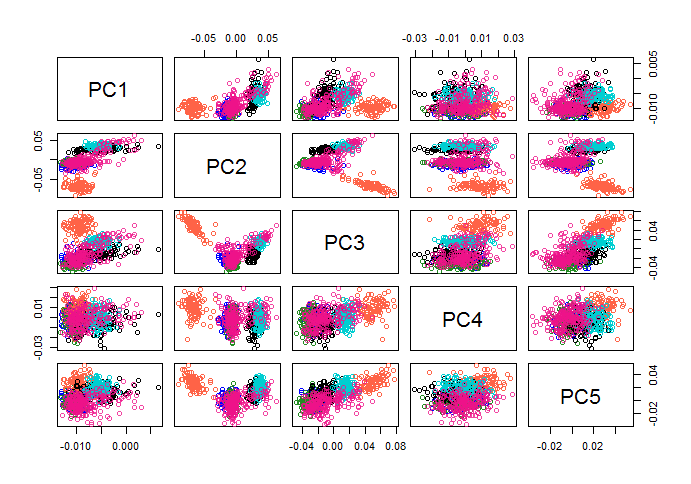
**

eigenvector$pop1kg[eigenvector$PC2 > 0.02 & eigenvector$PC3 > 0.05] <- "ol"

eigenvector$pop1kg[eigenvector$PC1 > 0.005] <- "ol"

eigenvector$pop1kg[eigenvector$PC4 > 0.1] <- "ol"

eigenvector$pop1kg[eigenvector$PC5 < -0.04] <- "ol"

eigenvector$pop1kg[eigenvector$PC3 > 0.05] <- "ol"

**KIRP**

**
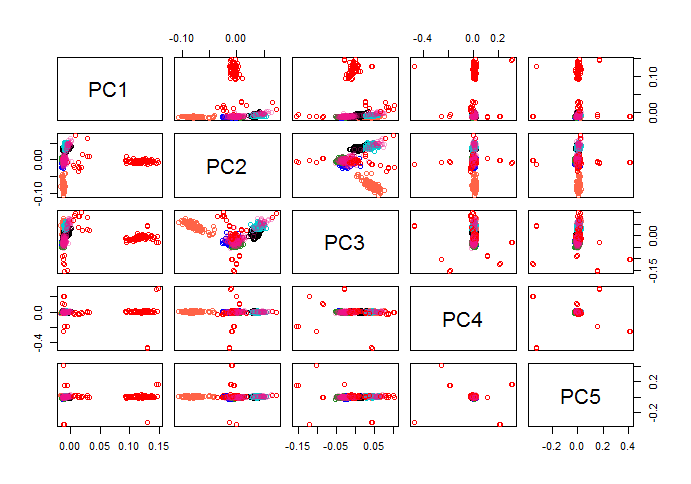
**

**
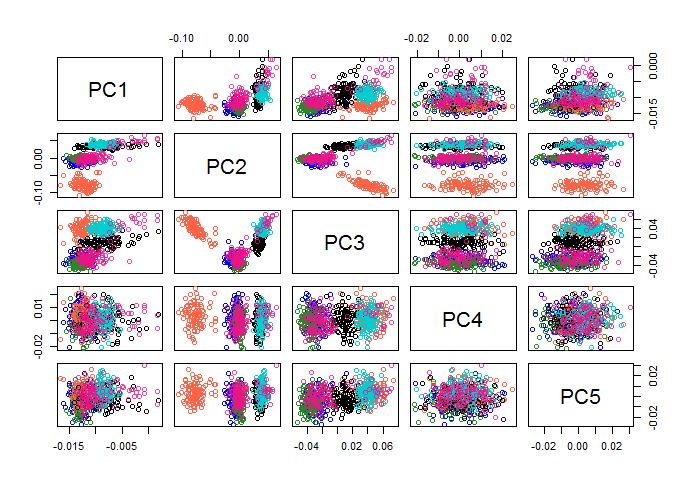
**

eigenvector$pop1kg[eigenvector$PC1 > 0.004] <- "ol"

eigenvector$pop1kg[eigenvector$PC4 > 0.1] <- "ol"

eigenvector$pop1kg[eigenvector$PC4 < -0.05] <- "ol"

eigenvector$pop1kg[eigenvector$PC3 < -0.07] <- "ol"

**LGG**

**
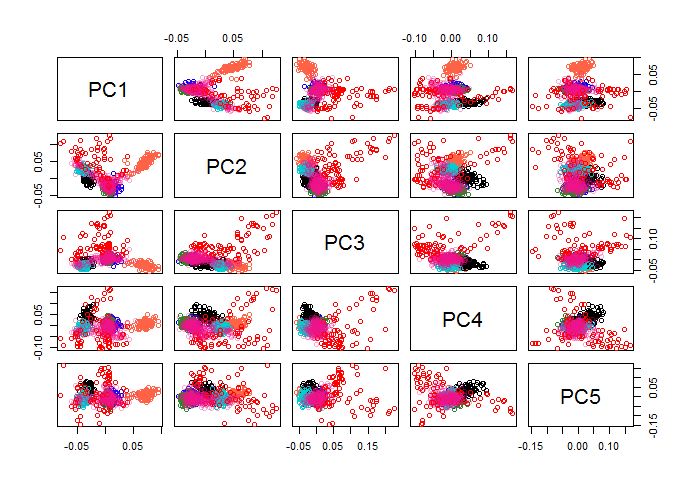
**

**
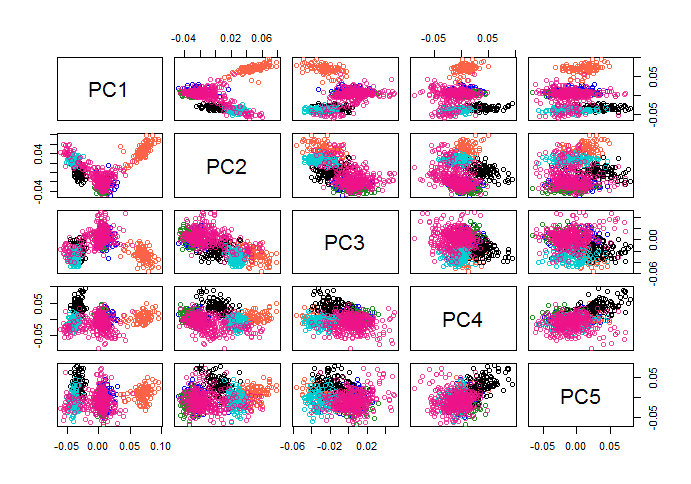
**

eigenvector$pop1kg[eigenvector$PC2 > 0 & eigenvector$PC3 > 0.02] <- "ol"

eigenvector$pop1kg[eigenvector$PC2 > 0.04 & eigenvector$PC3 > 0] <- "ol"

eigenvector$pop1kg[eigenvector$PC5 > 0.08] <- "ol"

eigenvector$pop1kg[eigenvector$PC2 > 0.07] <- "ol"

eigenvector$pop1kg[eigenvector$PC5 < -0.1] <- "ol"

eigenvector$pop1kg[eigenvector$PC3 > 0.05] <- "ol"

**LIHC**

**
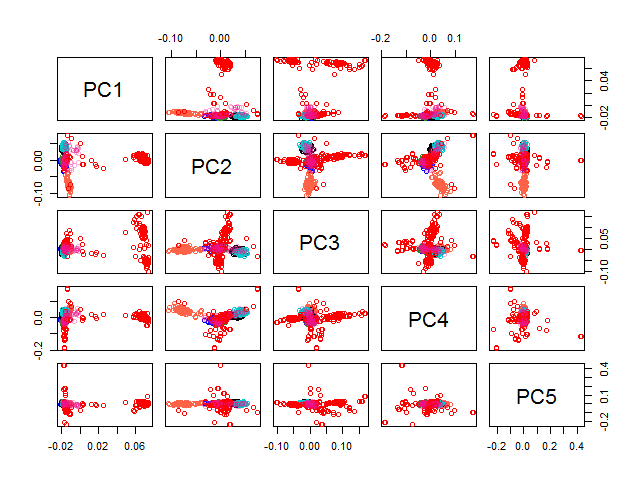
**

**
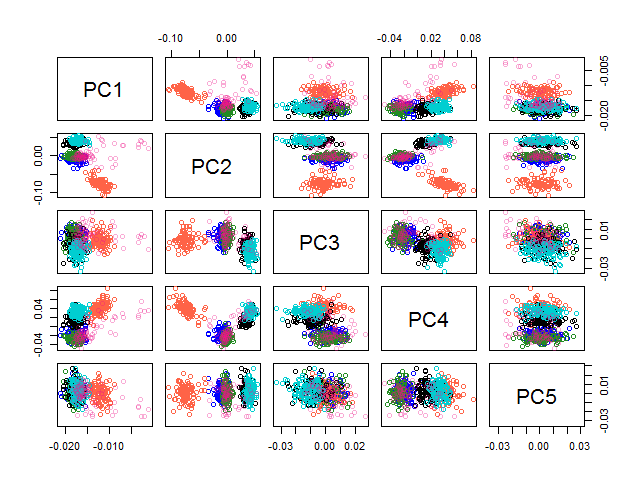
**

**LUAD**

**
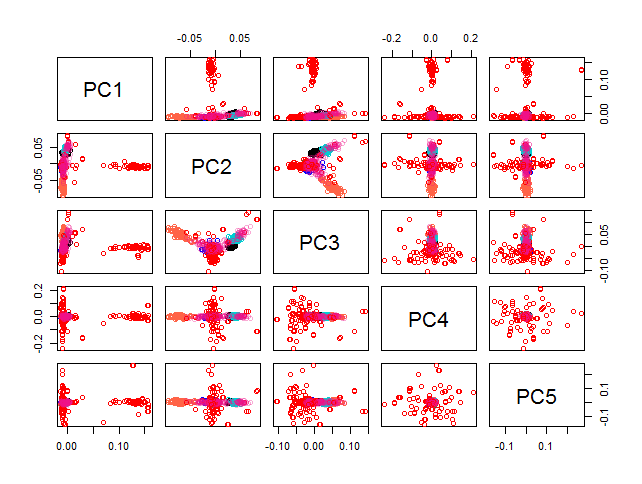
**

**
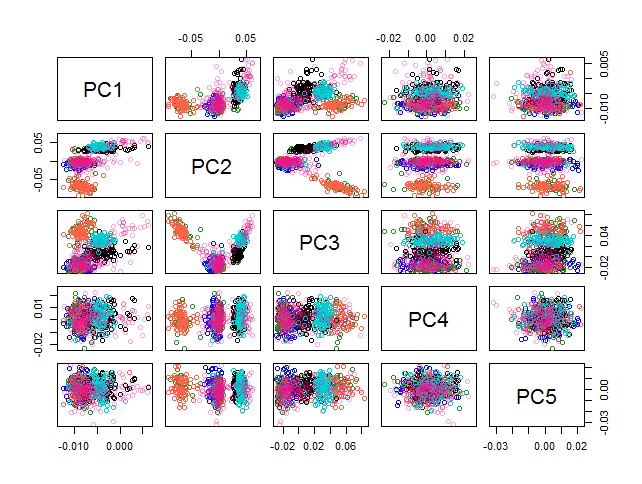
**

eigenvector$pop1kg[eigenvector$PC1 > 0.01] <- "ol"

eigenvector$pop1kg[eigenvector$PC5 < -0.04] <- "ol"

eigenvector$pop1kg[eigenvector$PC5 > 0.05] <- "ol"

eigenvector$pop1kg[eigenvector$PC4 > 0.035] <- "ol"

eigenvector$pop1kg[eigenvector$PC4 < -0.03] <- "ol"

eigenvector$pop1kg[eigenvector$PC3 < -0.028] <- "ol"

**LUSC**

**
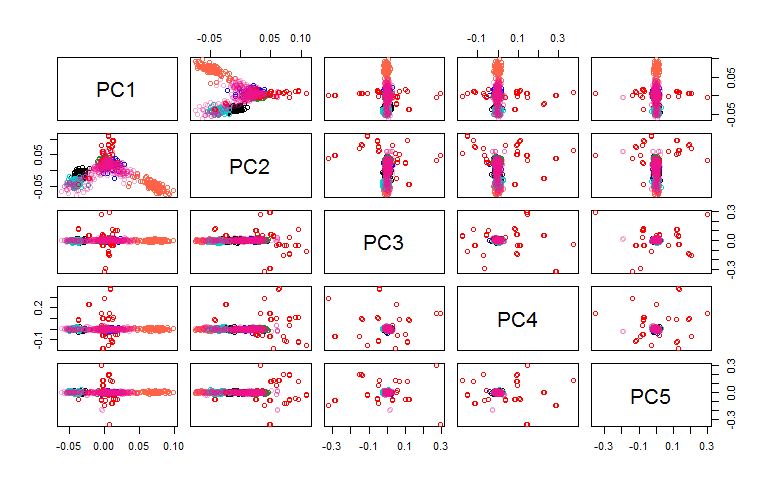
**

**
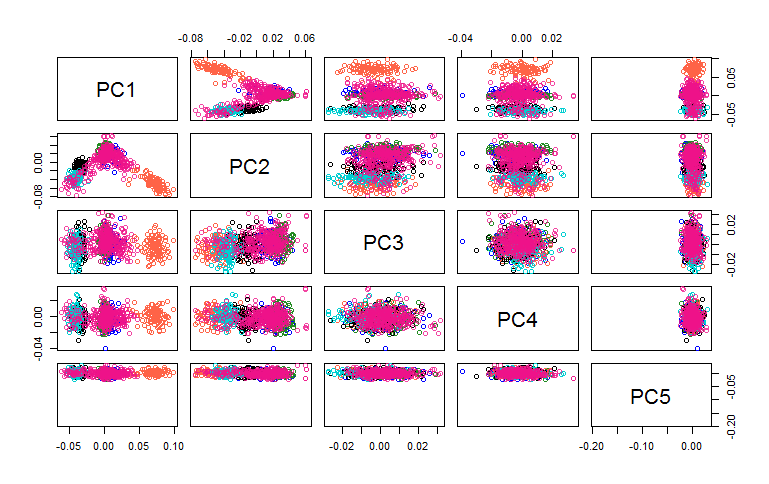
**

eigenvector$pop1kg[eigenvector$PC4 > 0.05] <- "ol"

eigenvector$pop1kg[eigenvector$PC4 < -0.05] <- "ol"

eigenvector$pop1kg[eigenvector$PC3 > 0.05] <- "ol"

eigenvector$pop1kg[eigenvector$PC3 < -0.07] <- "ol"

eigenvector$pop1kg[eigenvector$PC2 > 0.07] <- "ol"

**PAAD (no outliers)**

**
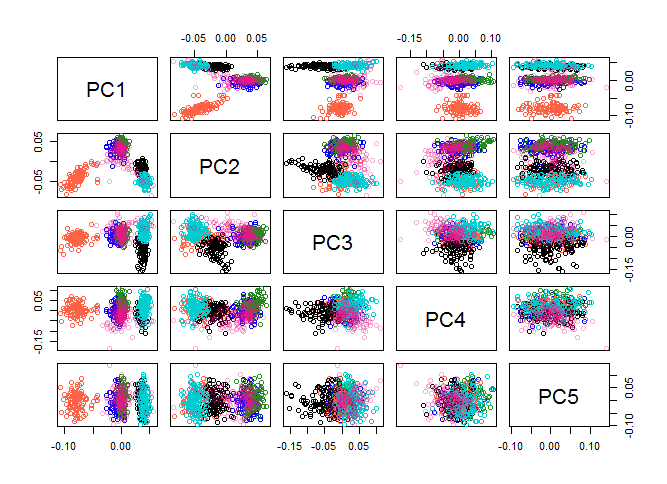
**

**PCPG (no outliers)**

**
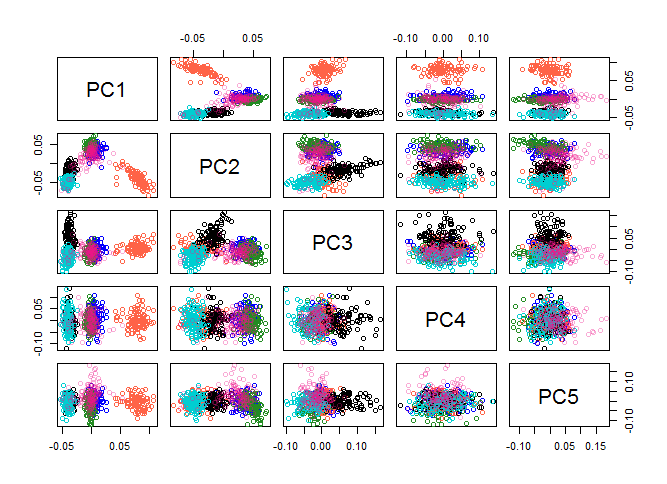
**

**PRAD**

**
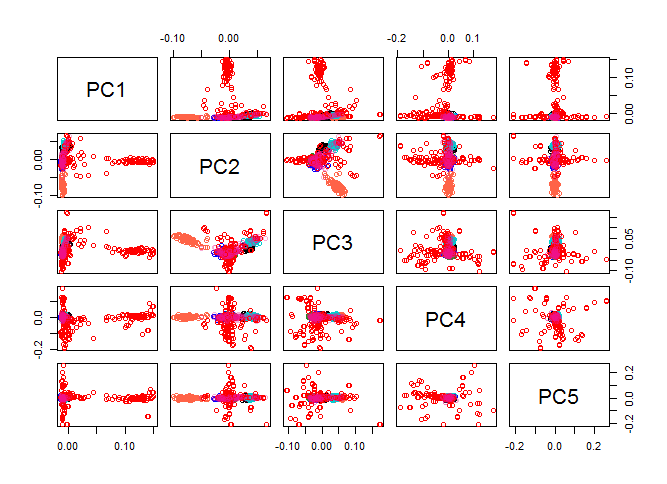
**

**
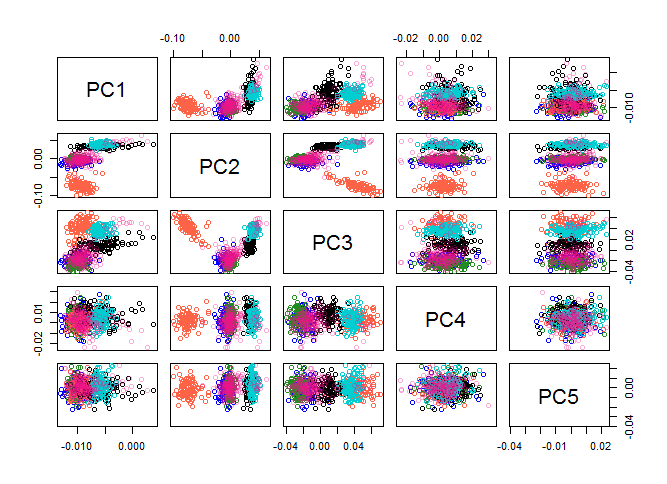
**

eigenvector$pop1kg[eigenvector$PC3 > 0.05 & eigenvector$PC1 > 0] <- "ol"

eigenvector$pop1kg[eigenvector$PC1 > -0.005 & eigenvector$PC2 < 0.01] <- "ol"

eigenvector$pop1kg[eigenvector$PC1 > 0.01] <- "ol"

eigenvector$pop1kg[eigenvector$PC5 > 0.1] <- "ol"

eigenvector$pop1kg[eigenvector$PC5 < -0.04] <- "ol"

eigenvector$pop1kg[eigenvector$PC4 > 0.05] <- "ol"

eigenvector$pop1kg[eigenvector$PC4 < -0.03] <- "ol"

eigenvector$pop1kg[eigenvector$PC3 < -0.05] <- "ol"

**READ**

**
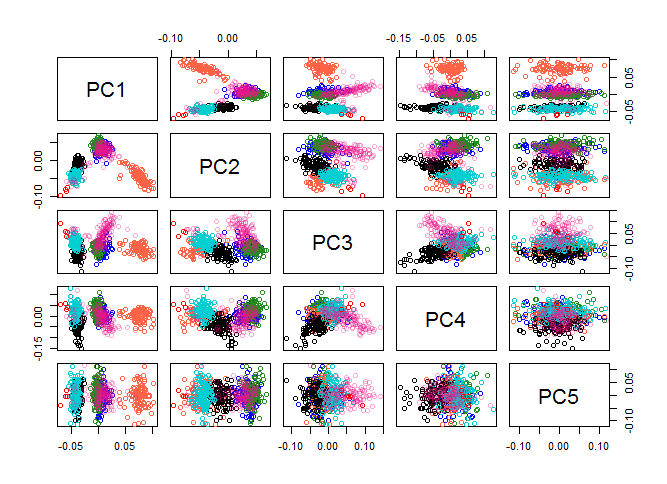
**

**
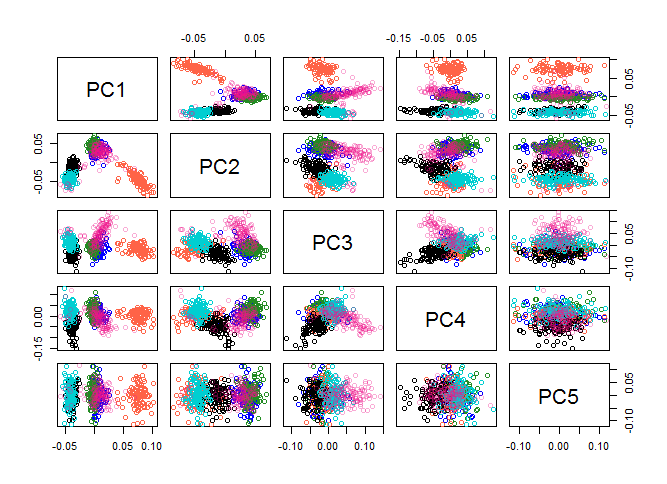
**

eigenvector$pop1kg[eigenvector$PC1 < -0.05 & eigenvector$PC2 < -0.075] <- "ol"

**SARC**

**
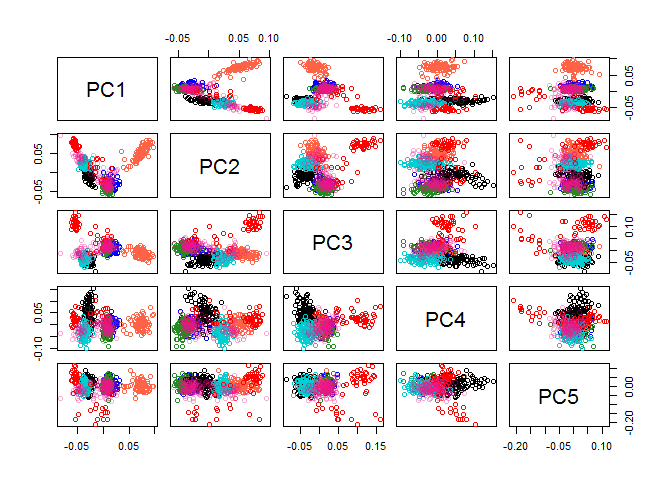
**

**
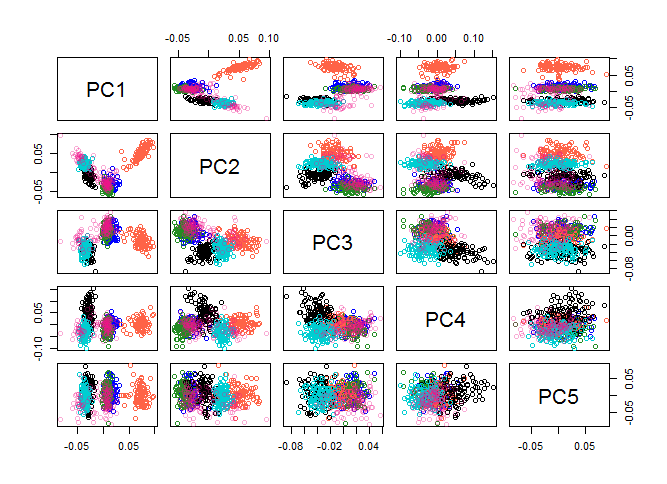
**

eigenvector$pop1kg[eigenvector$PC3 > 0.05 & eigenvector$PC2 > 0.0025] <- "ol"

eigenvector$pop1kg[eigenvector$PC5 < -0.1] <- "ol"

eigenvector$pop1kg[eigenvector$PC3 > 0.06] <- "ol"

**SKCM**

**
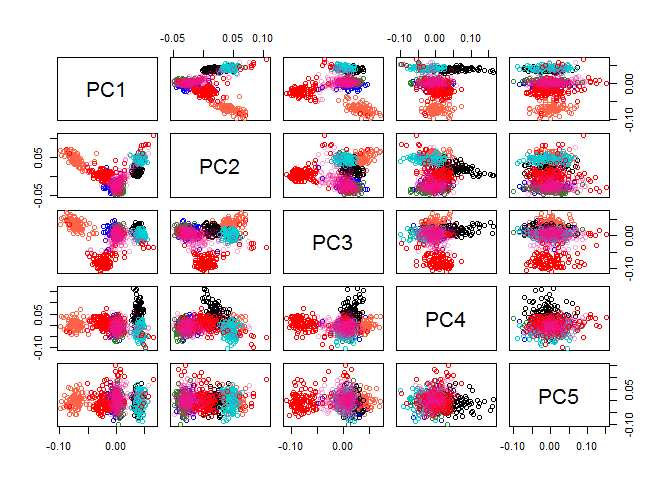
**

**
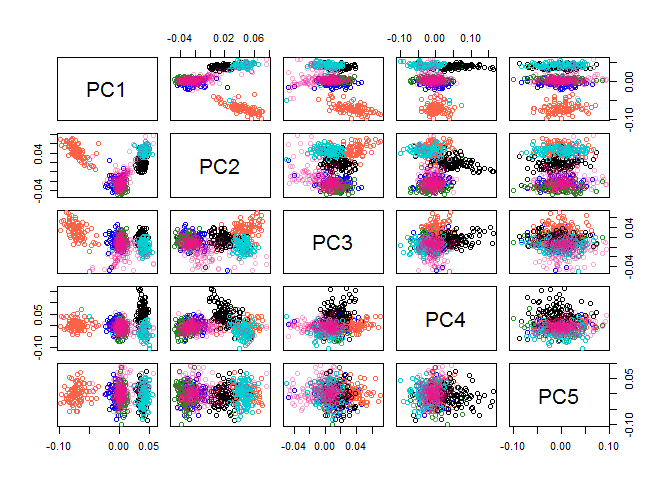
**

eigenvector$pop1kg[eigenvector$PC3 < -0.05] <- "ol"

eigenvector$pop1kg[eigenvector$PC4 < -0.025 & eigenvector$PC2 > 0.06] <- "ol"

eigenvector$pop1kg[eigenvector$PC5 > 0.1] <- "ol"

**STAD**

**
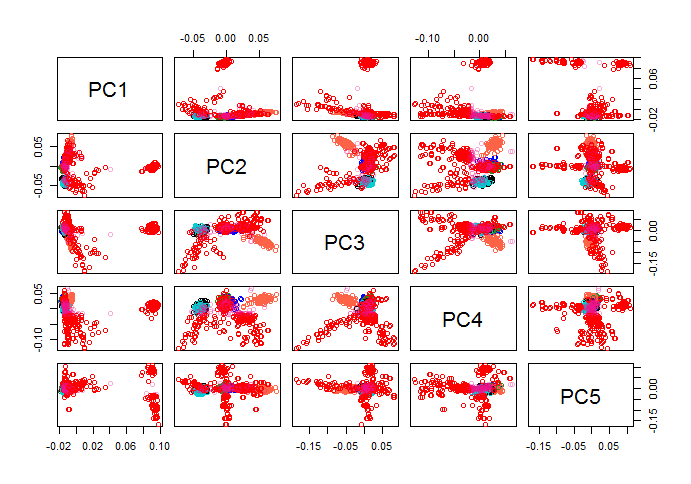
**

**
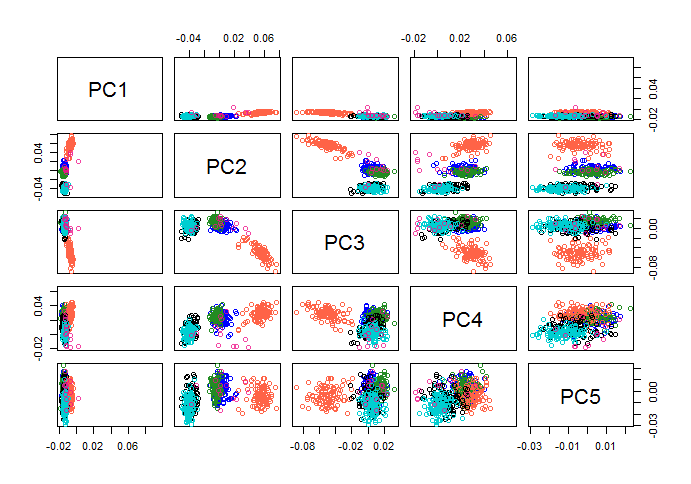
**

eigenvector$pop1kg[eigenvector$PC5 > 0.025] <- "ol"

eigenvector$pop1kg[eigenvector$PC5 < -0.03] <- "ol"

eigenvector$pop1kg[eigenvector$PC4 < -0.02] <- "ol"

eigenvector$pop1kg[eigenvector$PC3 > 0.04] <- "ol"

**TGCT (no outliers)**

**
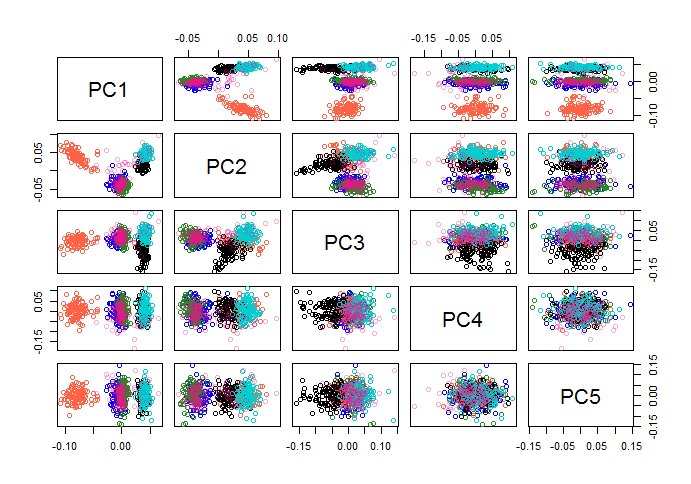
**

**THCA**

**
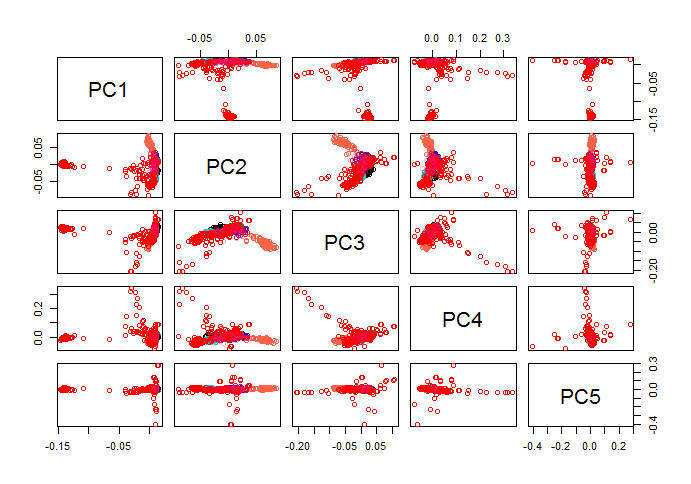
**

**
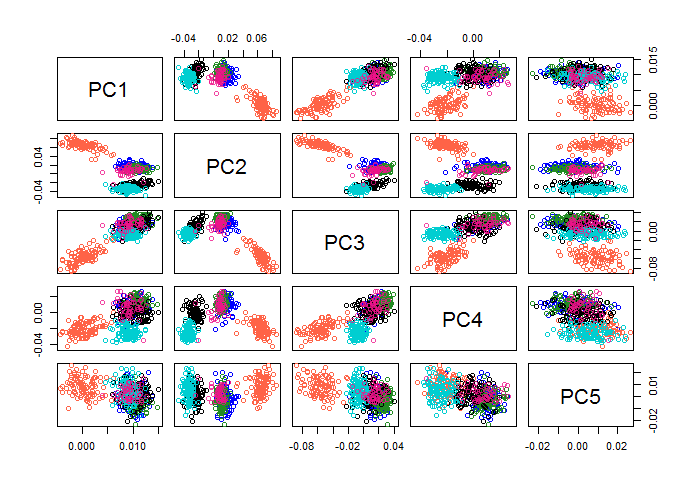
**

eigenvector$pop1kg[eigenvector$PC1 < 0.05 & eigenvector$PC2 < -0.03] <- "ol"

eigenvector$pop1kg[eigenvector$PC1 < -0.005] <- "ol"

eigenvector$pop1kg[eigenvector$PC5 > 0.015] <- "ol"

eigenvector$pop1kg[eigenvector$PC5 < -0.005] <- "ol"

eigenvector$pop1kg[eigenvector$PC4 > 0.03] <- "ol"

eigenvector$pop1kg[eigenvector$PC3 > 0.05] <- "ol"

eigenvector$pop1kg[eigenvector$id2 %in% hap.ids$ID[hap.ids$Population == "CEU"]] <- "CEU"

eigenvector$pop1kg[eigenvector$id2 %in% hap.ids$ID[hap.ids$Population == "TSI"]] <- "TSI"

eigenvector$pop1kg[eigenvector$id2 %in% hap.ids$ID[hap.ids$Population == "GBR"]] <- "GBR"

eigenvector$pop1kg[eigenvector$id2 %in% hap.ids$ID[hap.ids$Population == "FIN"]] <- "FIN"

eigenvector$pop1kg[eigenvector$id2 %in% hap.ids$ID[hap.ids$Population == "IBS"]] <- "IBS"

**UCEC**

**
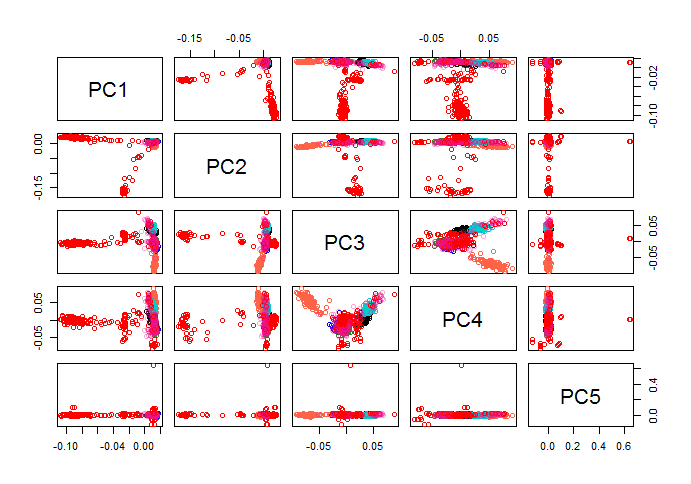
**

**
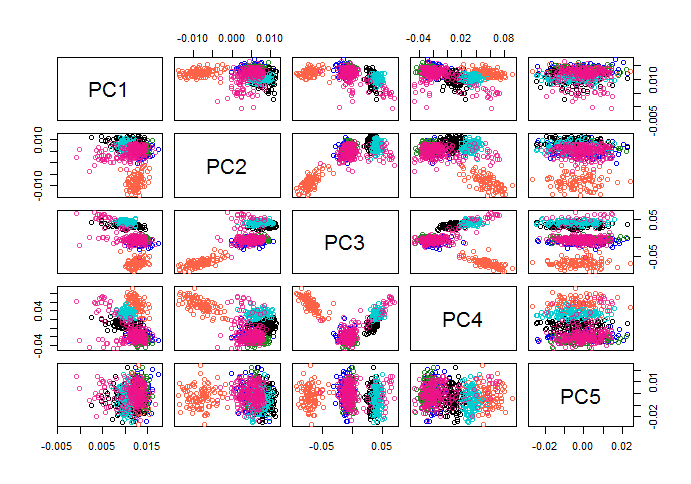
**

eigenvector$pop1kg[eigenvector$PC4 < -0.05 & eigenvector$PC5 < -0.02] <- "ol"

eigenvector$pop1kg[eigenvector$PC1 < -0.005] <- "ol"

eigenvector$pop1kg[eigenvector$PC5 > 0.015] <- "ol"

eigenvector$pop1kg[eigenvector$PC2 < -0.018] <- "ol"

eigenvector$pop1kg[eigenvector$PC5 < -0.04] <- "ol"
