# Supplementary material for: iModEst: disentangling -omic impacts on gene expression variation across genes and tissues
Source: NAR Genom Bioinform. 2025 Mar 4;7(1):lqaf011. doi: 10.1093/nargab/lqaf011 (PMC11879402; doi:10.1093/nargab/lqaf011)
Supplement: lqaf011_Supplemental_Files [file lqaf011_supplemental_files.zip › SuppFile2_iModEst Interactive summary.docx]

**Supplementary File 1**

This supplementary file briefly describes how to interact with iModEst to look at a single gene. We will use the *TP53* gene as a use-case. The majority of iModEst’s interactivity can be found in the paired web application, <https://imodesttool.com>.

## Plotting the regulatory modalities of a gene of interest

The first page, “By Tissue”, summarizes *TP53*’s gene expression regulation across each regulatory modality and cancer.


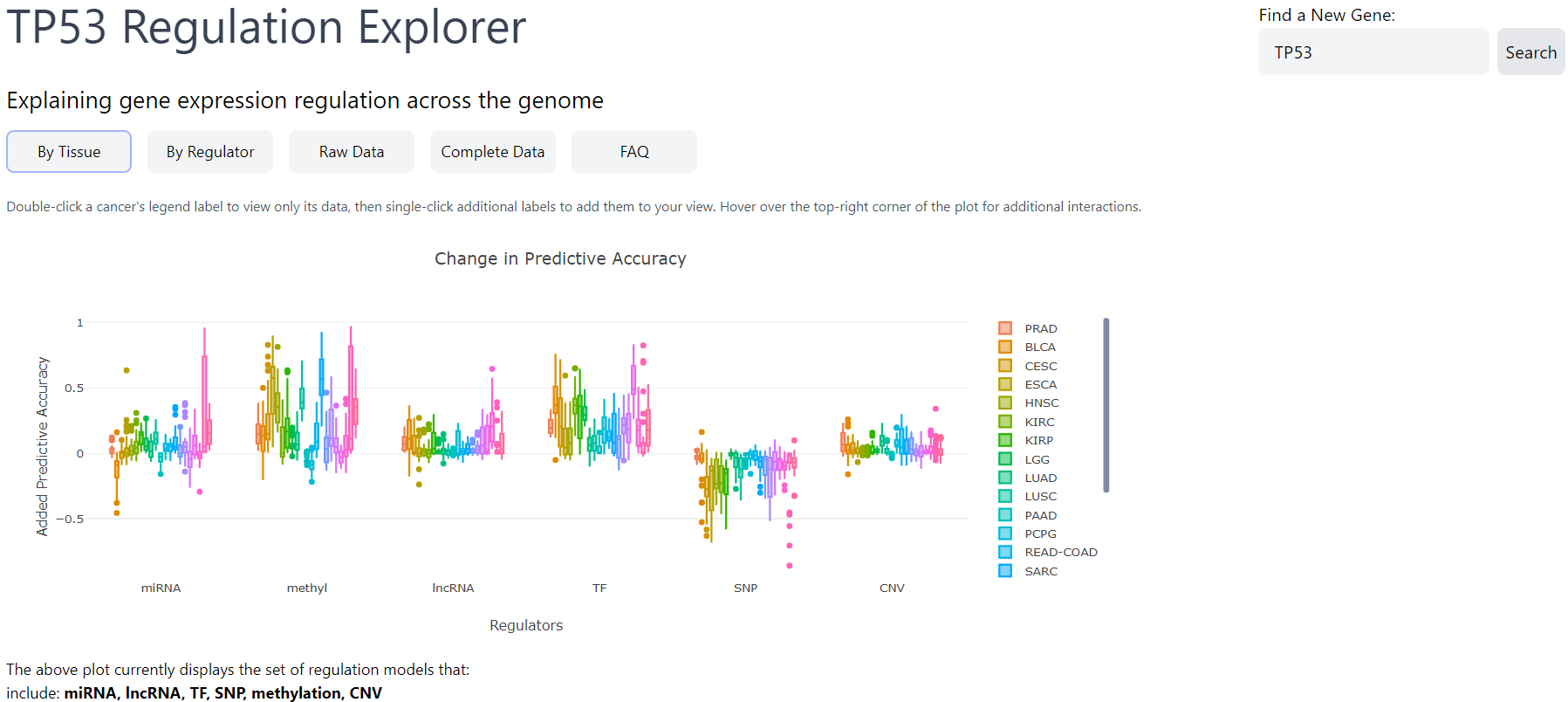


To visualize the predictive gain of each regulator and cancer on your gene can be visualized by hovering over the barplot.


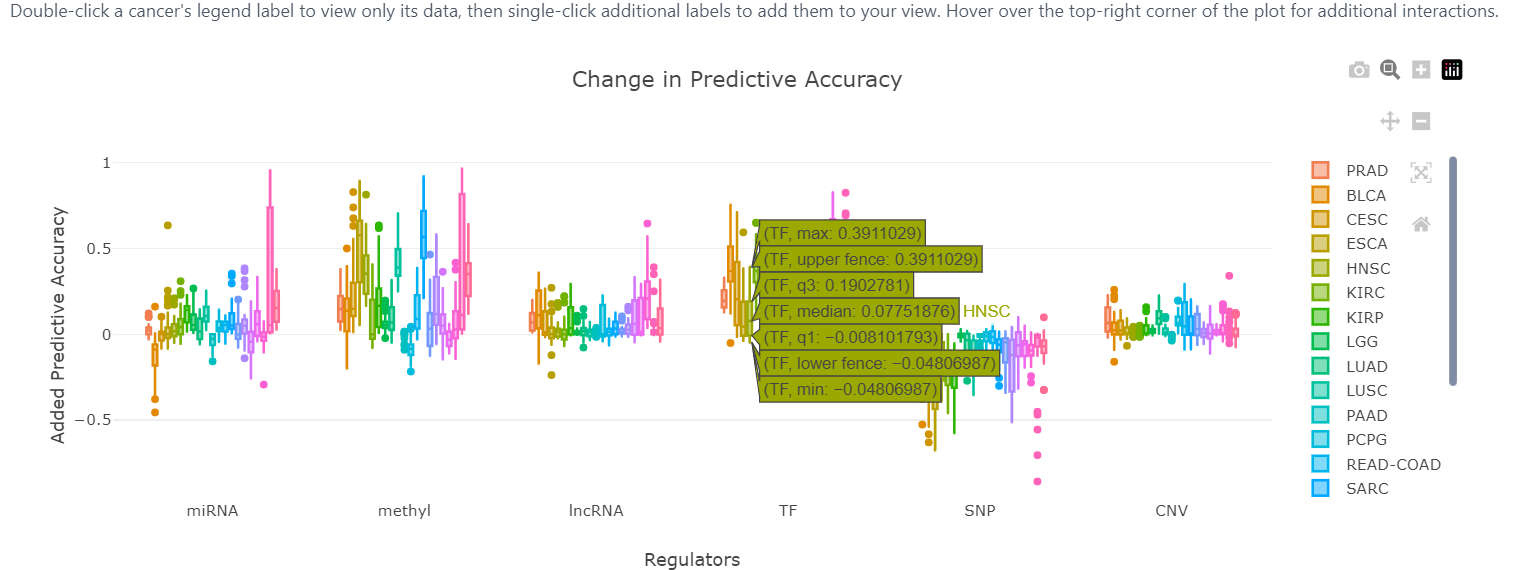


Regulation of the same gene can be reloaded by clicking the “Generate Visualization for Regulatory Effects” bar. In this example, we have re-computed the regulation of *TP53* while controlling for TFs within the model. In this example, controlling for TF regulation increases the impact of miRNA’s on *TP53* expression in BRCA.


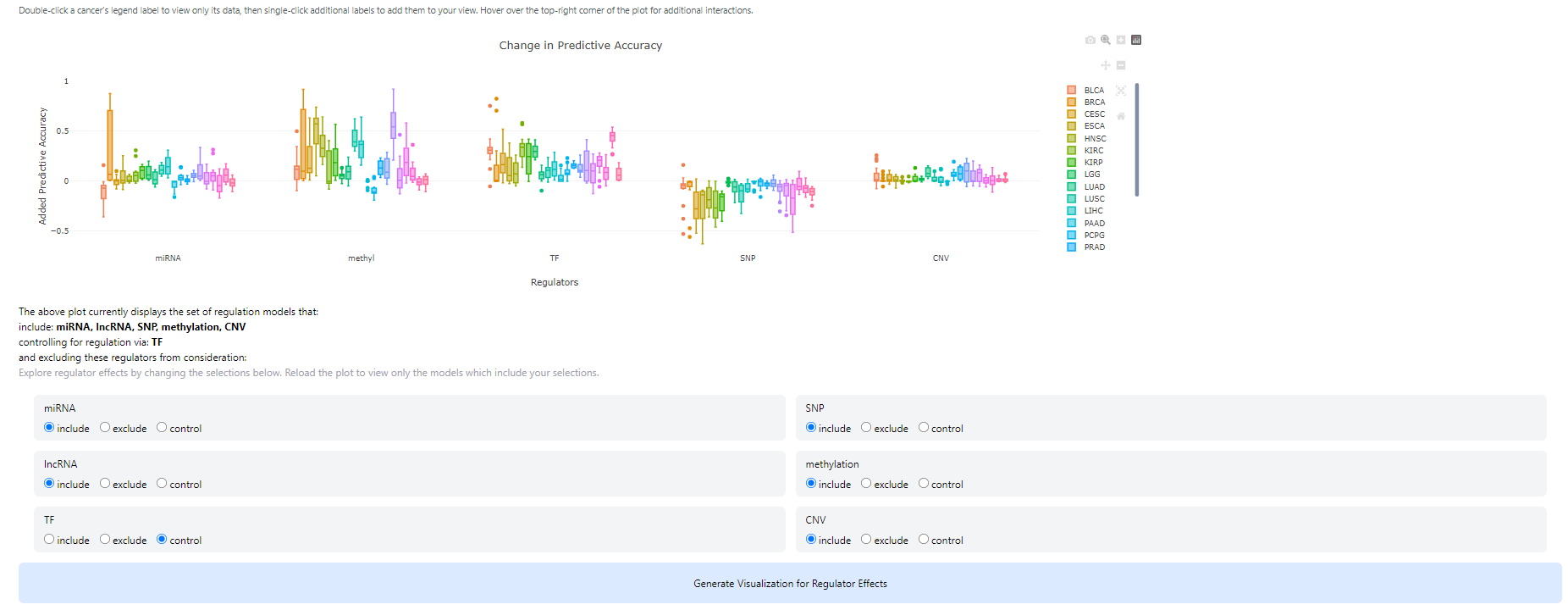


## Visualizing the coefficients of the regulatory molecules impacting your gene of interest

iModEst does not only compute which regulatory modality is impacting gene expression, but it also displays which specific molecules (TF genes, mature miRNAs, lncRNA genes, CNVs, SNVs, and methylation CpGs) impact gene expression. The impact of these specific molecules are measured by the coefficients of each regulator computed from the elastic net regression in each model. These regulators can be plotted by the “By Regulator” tab.


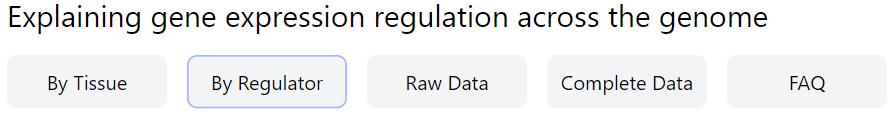


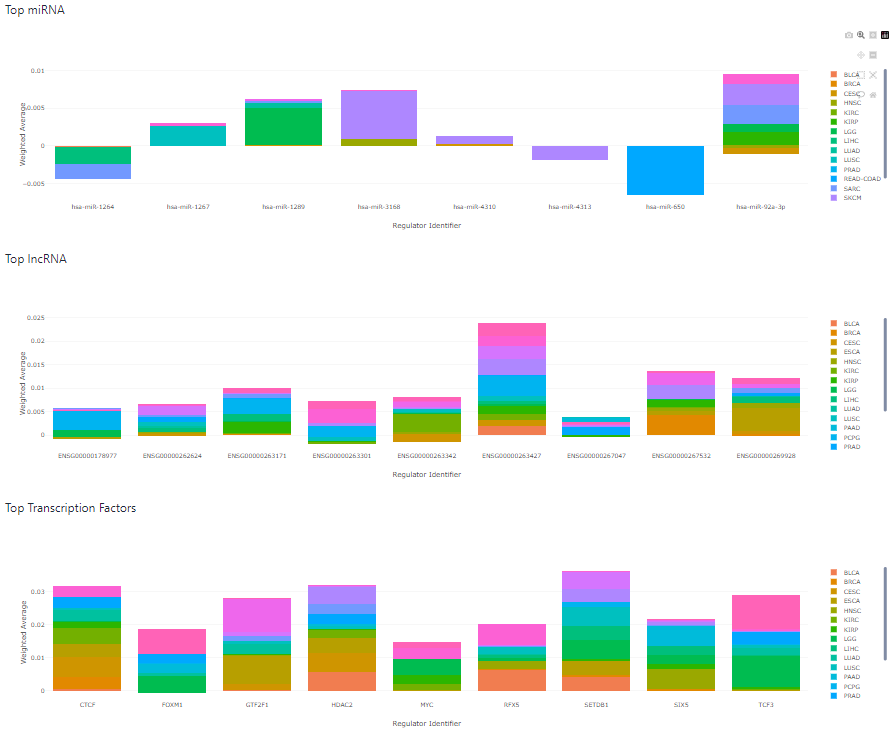


In this example, we can see that the CTCF transcription factor contributes to the TF regulation in *TP53* expression across multiple cancers. Like with the predictive gain of each regulator, we can visualize the coefficient of each regulator upon *TP53* by hovering over the gene.


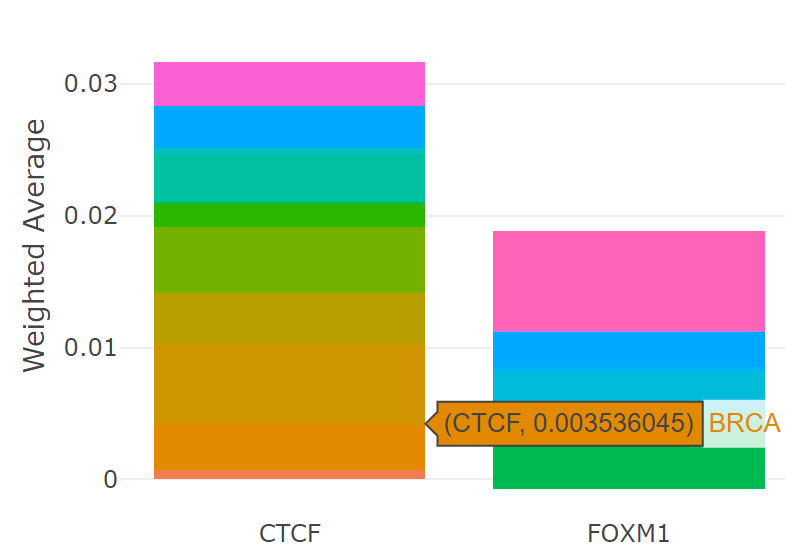


### Plotting SNPs and methylation

A coefficient was computed for each CpG and SNP in relationship to each gene and cancer, providing the potential of thousands of coefficients for each gene. Accordingly, we generated bigwig files of these coefficients so that they can be plotted on the UCSC genome browser. To plot these coefficients, load the two data hubs onto the hg19 genome by copying each link into the URL:

<http://wilsonlab.org/public/iModEst/methyl_hub/hub.txt>

<http://wilsonlab.org/public/iModEst/SNP_hub/hub.txt>


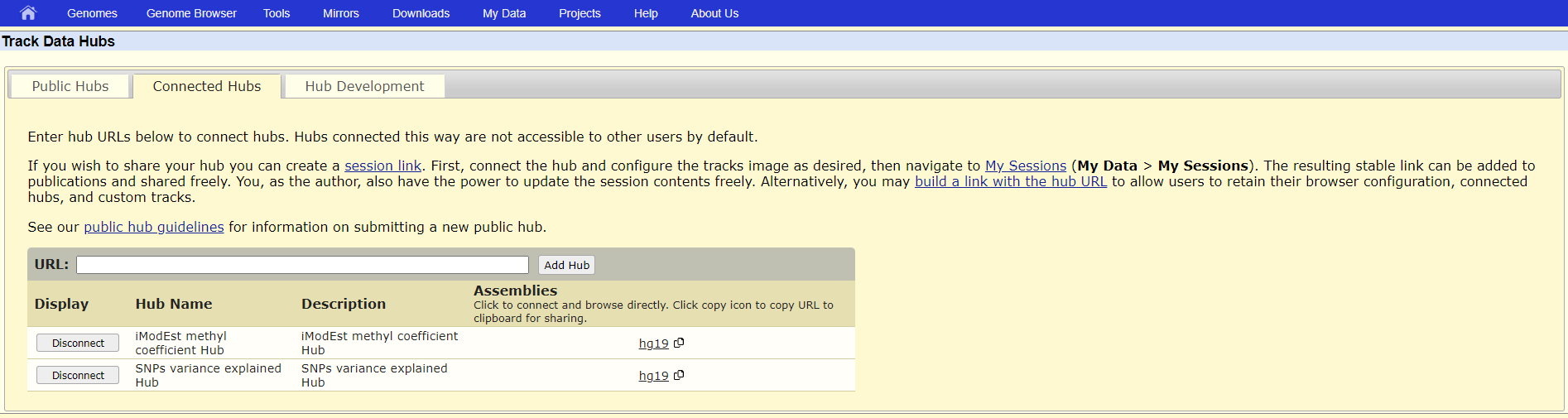


Below is a genome browser screenshot of the 200Kb surrounding the *TP53* gene with coefficients calculated in BRCA. Histograms in a positive direction are coefficients with a positive association on gene expression (i.e., the presence of the SNP or CpG is associated with a higher expression), while histograms in a negative direction are coefficients with a negative expression.


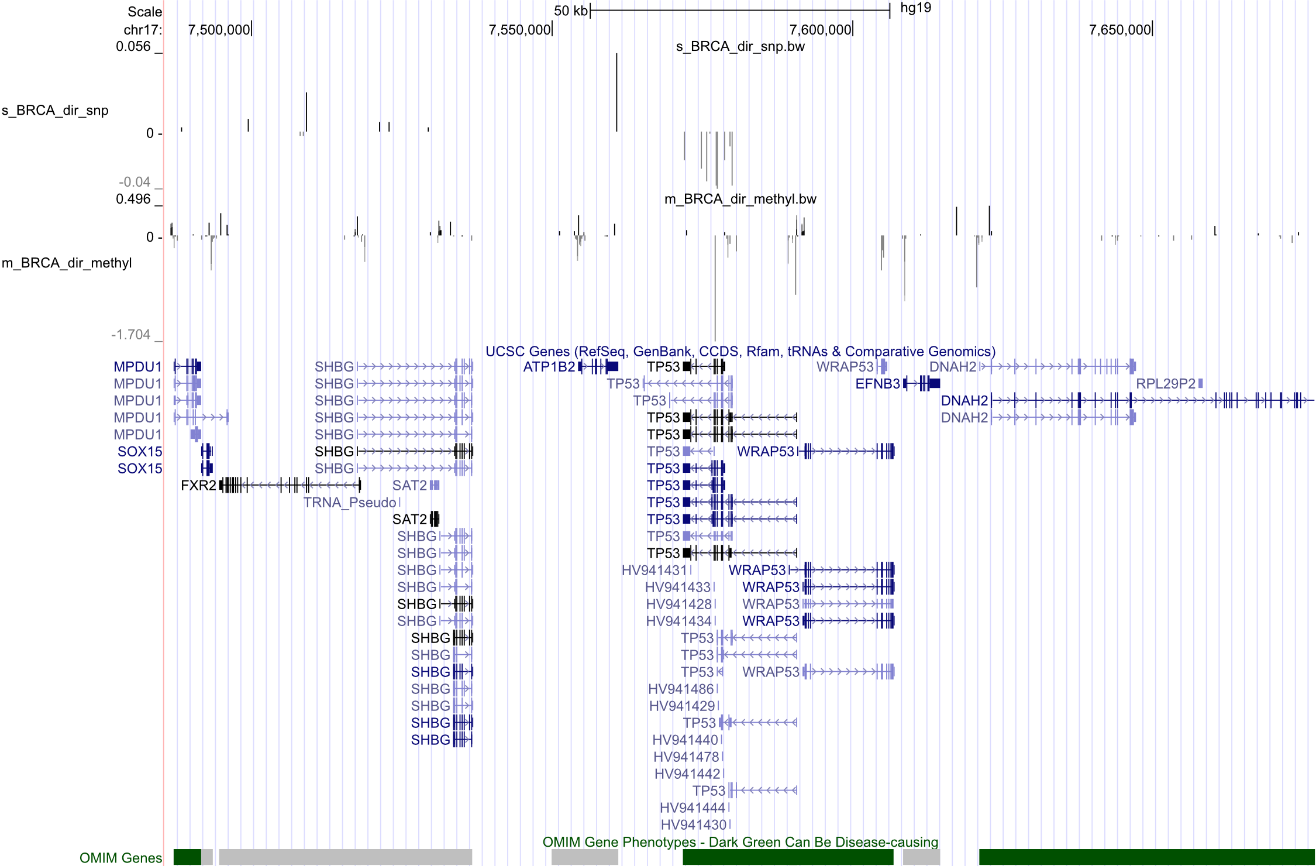


Adding additional cancers can be found in the track data hub. Select “magnitude” instead of “dir” if disinterested in the directionality of the coefficient.


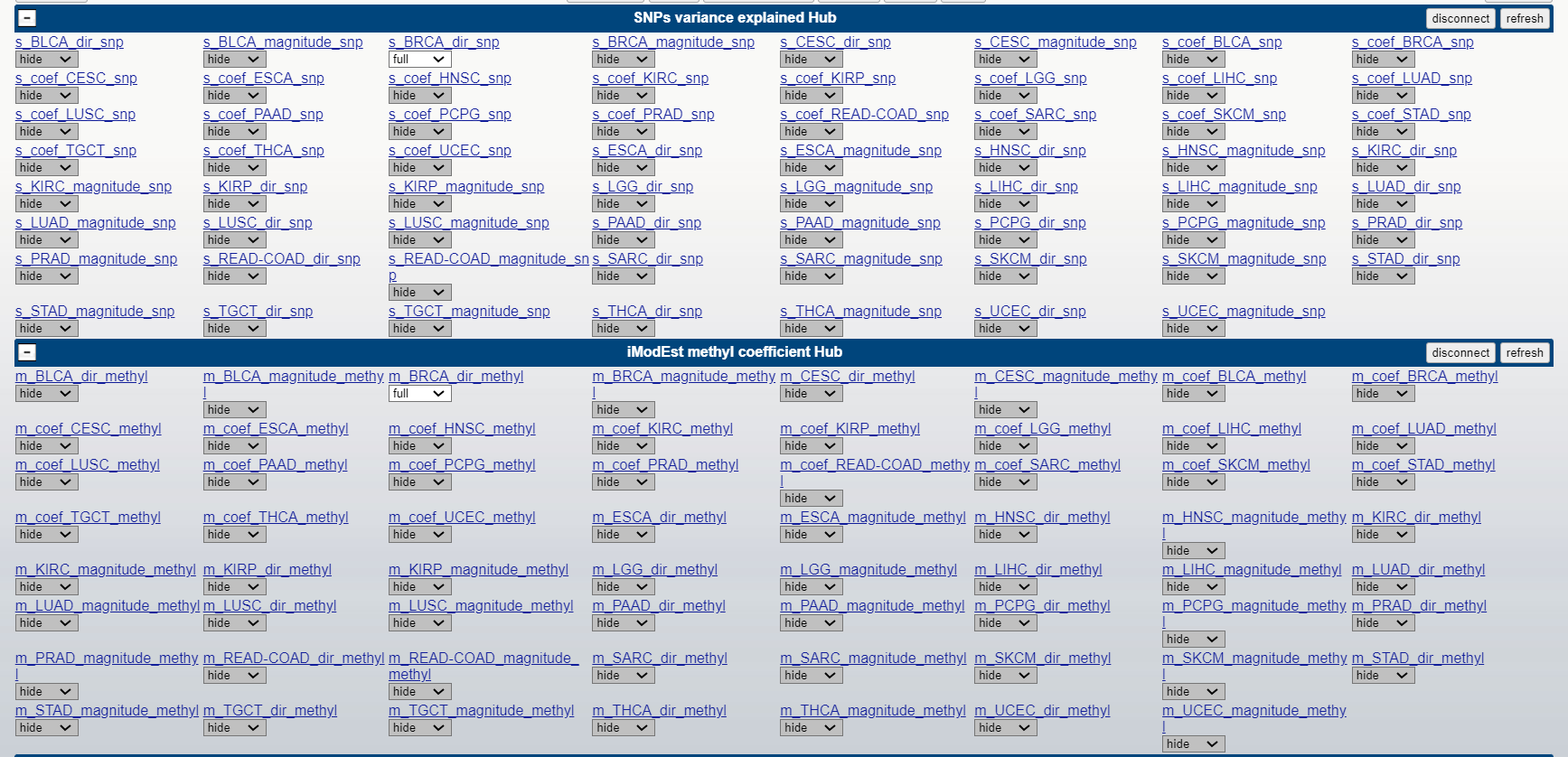


## Interacting with raw and processed data stored in iModEst

The values of the predictive gain of each regulatory class (i.e., TF, methylation, miRNA, SNP, and CNV PRESS R^2^ growth) and the impact of each regulatory molecule (miRNA, lncRNA, and TF coefficients) on *TP53* expression can be found in the “Raw Data” tab.


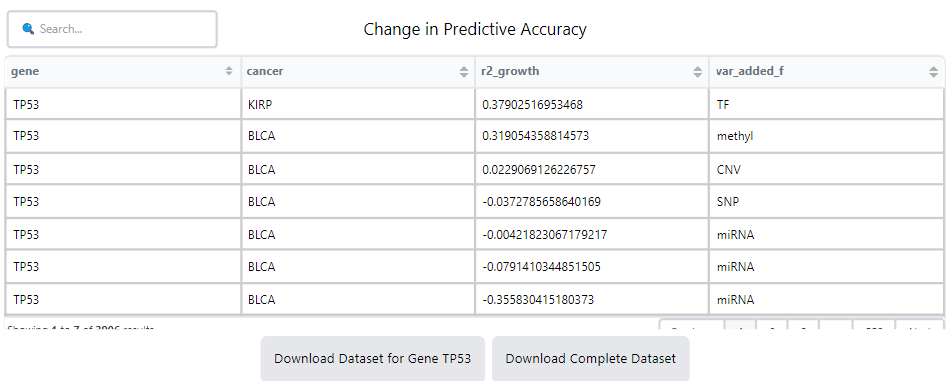


For future meta-analysis and the impact of regulation across many genes, processes data may be downloaded with under the “Complete Data” tab.
